# Xanthohumol overcomes osimertinib resistance via governing ubiquitination-modulated Ets-1 turnover

**DOI:** 10.1038/s41420-024-02220-y

**Published:** 2024-10-28

**Authors:** Ying Ma, Ruirui Wang, Jinzhuang Liao, Pengfei Guo, Qiang Wang, Wei Li

**Affiliations:** 1 Department of Radiology, The Third Xiangya Hospital of Central South University, Changsha, Hunan People’s Republic of China; 2 NHC key laboratory of translantional research on transplantation medicine, Department of Transplant Surgery, The Third Xiangya Hospital of Central South University, Changsha, Hunan People’s Republic of China

**Keywords:** Cancer therapy, Cell biology

## Abstract

Non-small cell lung cancer (NSCLC) is a prevalent and fatal malignancy with a significant global impact. Recent advancements have introduced targeted therapies like tyrosine kinase inhibitors (TKIs) such as osimertinib, which have improved patient outcomes, particularly in those with EGFR mutations. Despite these advancements, acquired resistance to TKIs remains a significant challenge. Hence, one of the current research priorities is understanding the resistance mechanisms and identifying new therapeutic targets to improve therapeutic efficacy. Herein, we identified high expression of c-Met in osimertinib-resistant NSCLC cells, and depletion of c-Met significantly inhibited the proliferation of osimertinib-resistant cells and prolonged survival in mice, suggesting c-Met as an attractive therapeutic target. To identify effective anti-tumor agents targeting c-Met, we screened a compound library containing 641 natural products and found that only xanthohumol exhibited potent inhibitory effects against osimertinib-resistant NSCLC cells. Moreover, combination treatment with xanthohumol and osimertinib sensitized osimertinib-resistant NSCLC cells to osimertinib both in vitro and in vivo. Mechanistically, xanthohumol disrupted the interaction between USP9X and Ets-1, and inhibited the phosphorylation of Ets-1 at Thr38, promoting its degradation, thereby targeting the Ets-1/c-Met signaling axis and inducing intrinsic apoptosis in osimertinib-resistant NSCLC cells. Overall, the research highlights the critical role of targeting c-Met to address osimertinib resistance in NSCLC. By demonstrating the efficacy of xanthohumol in overcoming resistance and enhancing therapeutic outcomes, this study provides valuable insights and potential new strategies for improving the clinical management of NSCLC.

## Facts

- Non-small cell lung cancer (NSCLC) is a globally prevalent and deadly malignancy.
- Osimertinib-based resistance is one of the main reasons for treatment failure in advanced NSCLC patients.
- Overcoming osimertinib resistance can benefit patients with NSCLC harboring EGFR activation mutations.
- Xanthohumol, a natural compound, has inhibitory effects on a wide range of tumors. However, its effect on osimertinib-resistant NSCLC cells is unclear.

## Introduction

Lung cancer remains the leading cause of cancer-related mortality worldwide, with non-small cell lung cancer (NSCLC) being the most prevalent histopathological subtype [1]. Standard treatments for NSCLC encompass surgery, radiotherapy, targeted biological therapy, and immunotherapy [1, 2]. Advances in precision medicine and biotechnology have significantly enhanced outcomes for NSCLC patients through the implementation of molecularly targeted therapies [3, 4]. Recent research has identified key carcinogenic drivers in NSCLC, particularly activating mutations in the epidermal growth factor receptor (EGFR) gene, which confer sensitivity to EGFR tyrosine kinase inhibitors (TKIs) such as erlotinib, gefitinib, and osimertinib [5, 6]. Among them, osimertinib has become a first-line standard of treatment strategy for advanced NSCLC patients harboring EGFR activation mutations [7]. Despite initial high response rates to osimertinib, nearly all patients inevitably developed acquired resistance via constitutive activation of various downstream signals, resulting in treatment failure [8, 9]. Therefore, this research aims to elucidate the mechanisms underlying osimertinib resistance, highlighting its significance in the development of new targeted agents. Such advancements are crucial for improving treatment outcomes for NSCLC patients with EGFR-activating mutations, ultimately enhancing clinical efficacy and patient quality of life.

The MET gene-encoded oncoprotein, c-Met, plays a crucial role in various cellular processes, including cell proliferation, migration, survival, and angiogenesis [10]. Emerging research suggests that upregulation of c-Met is frequently observed in various human tumors, including breast cancer, colorectal cancer, hepatocellular carcinoma, and head and neck squamous cell carcinoma [11–13]. This upregulation closely correlates with radio/chemoresistance, metastasis, the establishment of tumor immunosuppressive microenvironment, metabolic dysregulation, and unfavorable prognosis [12, 14–16]. Moreover, dysregulation of the c-Met pathway has been strongly implicated in cancer progression [17, 18], rendering it an attractive target for therapeutic intervention. Thus, revealing the mechanism of c-Met overexpression is critical for discovering the effective therapeutic approach for osimertinib-resistant NSCLC.

ETS proto-oncogene 1 (Ets-1) belongs to the ETS transcription factor family, characterized by its conserved ETS domain responsible for DNA binding [19]. Increasing studies have highlighted the involvement of Ets-1 in multiple cancer development and progression. Aberrant expression or activation of c-Met can disrupt gene expression patterns linked to oncogenesis [20–23]. Moreover, Ets-1 has been implicated in activating c-Met expression in melanoma and hepatocellular carcinoma cells, and its level correlates with poor prognosis [24–26]. Nevertheless, the potential impact of Ets-1 on osimertinib resistance in NSCLC remains elusive.

Xanthohumol, a prenylated flavonoid compound found in hops (Humulus lupulus) [27], commonly used in brewing beer, has garnered attention in emerging research for its diverse pharmacological activities, including anti-fungal, anti-viral, anti-bacterial, anti-inflammatory, anti-malarial, and anti-aging, anti-diabetes, anti-platelet, lipid-lowering and cardioprotective properties [28, 29]. Additionally, studies have explored its potential anti-cancer effects, demonstrating inhibition of growth in various cancer cell types such as oral squamous cell carcinoma, nasopharyngeal carcinoma, and breast cancer in preclinical studies [30–32], and preventing cancer initiation and progression by modulating multiple signaling pathways involved in cell proliferation, apoptosis, and metastasis [33, 34]. Moreover, xanthohumol has been implicated in enhancing the effectiveness of radiotherapy and chemotherapy against cancer [32, 35]. However, its impact on osimertinib-resistant NSCLC cells remains unclear, and its underlying mechanism has yet to be elucidated.

In our current study, we explored the effect of xanthohumol on the sensitivity of osimertinib-resistant NSCLC cells, discovering that the Ets-1/c-Met axis is pivotal in facilitating osimertinib resistance in NSCLC cells. Moreover, we demonstrated that xanthohumol effectively targets and inhibits this axis, offering a promising strategy to overcome osimertinib resistance.

## Results

### c-Met is essential for maintaining the tumorigenicity of osimertinib-resistant NSCLC

**Fig. 1:**
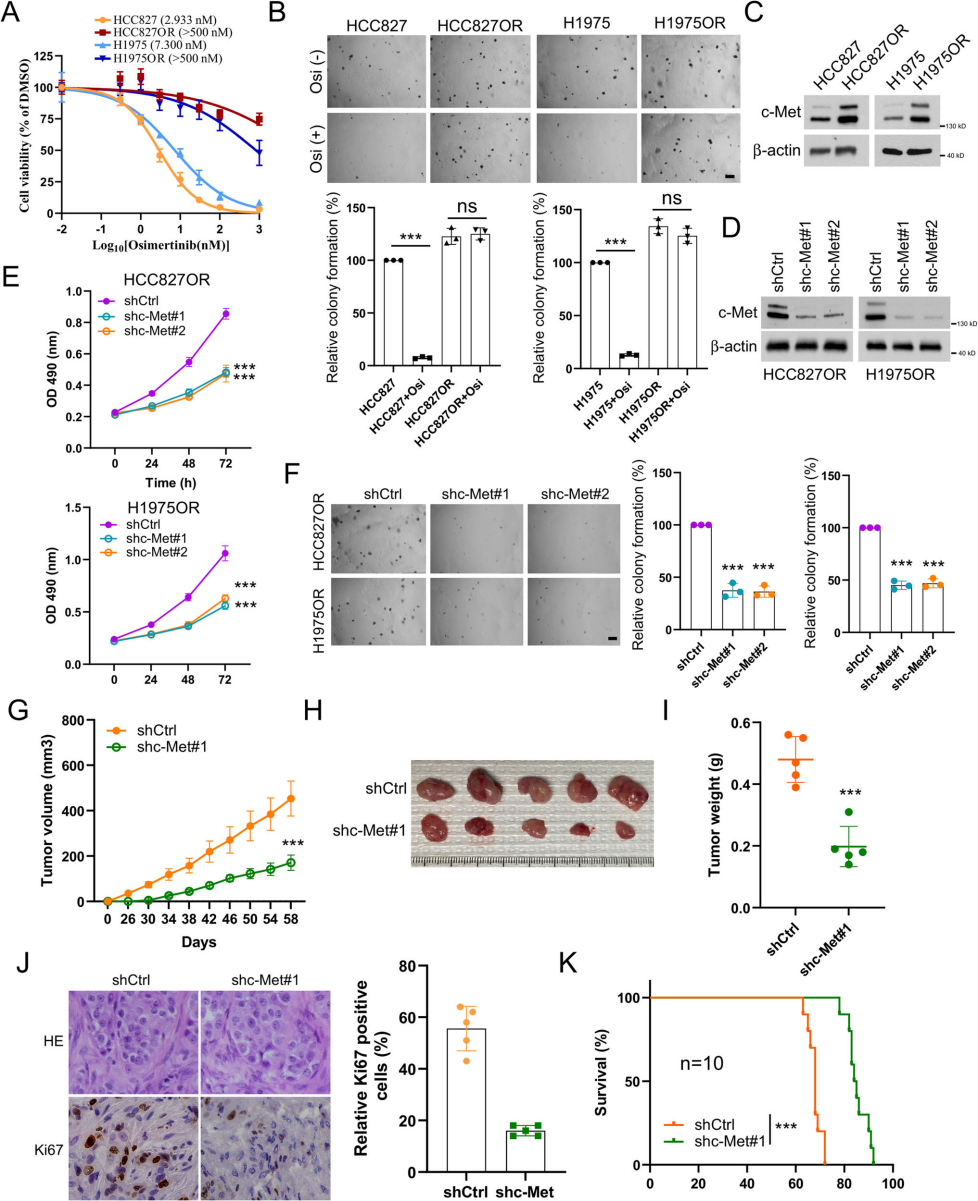
c-Met knockdown inhibits malignant phenotypes in osimertinib-resistant NSCLC cell lines. **A** MTS analysis of cell viability of HCC827/HCC827OR and H1975/H1975OR cells treated with osimertinib for 72 h. **B** Soft agar clone formation assay to analyze the colony-forming ability of HCC827/HCC827OR and H1975/H1975OR cells after 24 h of treatment with osimertinib. Scale bar, 200 μm. ns indicates no statistical significance. ***P < 0.001. **C** Immunoblotting assay to detect the protein expression level of c-Met in NSCLC parental and drug-resistant cells. Stable cell lines of HCC827OR and H1975OR knocked down with c-Met were constructed, and the protein expression level of c-Met was analyzed by WB (**D**), cell viability was analyzed by MTS cell viability assay (**E**), and the soft agar clone formation assay colony forming ability was analyzed (**F**). Scale bar, 200 μm. ***P < 0.001. Xenograft tumor models were analyzed for the effect of c-Met knockdown on HCC827OR tumor volume (**G**), tumor mass (**H**), tumor weight (**I**), Ki67 expression level (**J**), and mouse survival (**K**). Scale bar, 25 μm. ***P < 0.001.

c-Met overexpression is closely associated with resistance to cancer therapy. To understand the importance of c-Met in osimertinib resistance in NSCLC, we first used osimertinib-resistant cell lines HCC827OR and H1975OR and osimertinib-sensitive cell lines HCC827 and H1975 to perform cell viability and soft agar assays. The results indicated that the HCC827OR and H1975OR cells with osimertinib treatment had significantly higher IC_50_ values compared to HCC827 and H1975 cells (Fig. 1A). Moreover, there was no significant difference in anchorage-independent growth before and after osimertinib treatment in HCC827OR and H1975OR cells. In contrast, the growth of HCC827 and H1975 cells was significantly inhibited by osimertinib treatment (Fig. 1B). We then examined c-Met expression levels in sensitive and resistant cell lines and found an upregulation of c-Met expression in the HCC827OR and H1975OR cells (Fig. 1C), suggesting that overexpression of c-Met may be an essential driver of osimertinib resistance in NSCLC. Therefore, we established stable cell lines with c-Met knockdown in the resistant cell lines HCC827OR and H1975OR (Fig. 1D). The results of cell viability and soft agar analysis showed that knockdown of c-Met significantly inhibited proliferation of the resistant cell lines (Fig. 1E, F). Furthermore, using shCtrl and shc-met HCC827OR cells, we established xenograft mouse models, which demonstrated that silencing c-Met significantly delayed in vivo tumor growth, as tumor volume, tumor size, and tumor weight were all smaller (Fig. 1G–I). Similarly, the data of IHC indicated that expression levels of Ki67 were significantly reduced in tumor tissue after silencing c-Met (Fig. 1J). Additionally, c-Met-deficient tumor-bearing mice had a more extended survival (Fig. 1K). These data suggest that c-Met is required to maintain the tumorigenicity of osimertinib-resistant NSCLC.

### Xanthohumol exerts a strong anti-tumor effect on osimertinib-resistant NSCLC cells

**Fig. 2:**
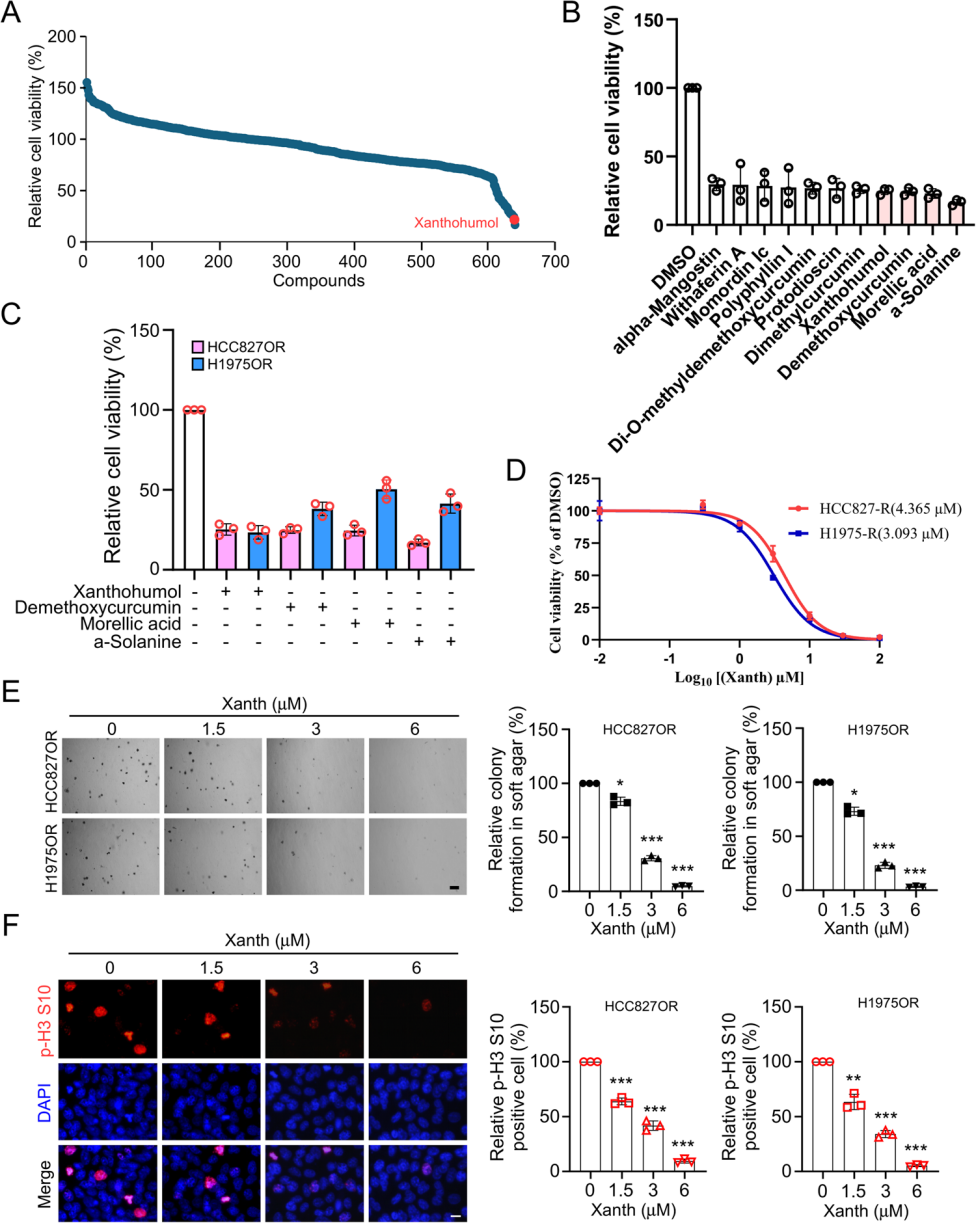
Xanthohumol inhibits osimertinib-resistant NSCLC cell lines. **A** Cell viability was determined by MTS assay to screen for natural compounds that down-regulate HCC827OR cell viability. The red dot is xanthohumol. **B** MTS assay to screen for drugs with 70% inhibition of HCC827OR cells. **C** MTS assay to analyze drugs with 70% inhibition of HCC827OR and H1975OR and identify this drug as xanthohumol. **D** MTS assay to determine the IC_50_ value of xanthohumol in HCC827OR/H1975OR. **E** Soft agar clone formation assay was performed to detect the colony-forming ability of HCC827OR and H1975OR cells after treatment with different concentrations of xanthohumol (0, 1.5, 3, and 6 μM) for 24 h. Scale bar, 200 μm. ***p < 0.001. **F** Immunofluorescence (IF) analysis of the phosphorylation level of histone H3 Ser10 in xanthohumol-treated HCC827OR cells. The left panel shows the IF staining image and the right panel shows the statistical analysis. Scale bar, 25 μM. **p < 0.01.***p < 0.001.

Numerous studies have demonstrated the remarkable anti-tumor effects of natural products, capable of reversing resistance to tumor radiotherapy and chemotherapy. Hence, we screened a customed natural product library containing 641 compounds and identified several compounds that significantly downregulated the cell viability of HCC827OR over 70% (The specific list of compounds can be found in Table S1), including alpha-mangostin, withaferin A, Di-O-methyldemethoxycurcumin, protodioscin, polyphyllin I, momordin Ic, dimethylcurcumin, xanthohumol (Xanth), demethoxycurcumin, morellic acid, and a-solanine (Fig. 2A, B). Among them, only four compounds, namely xanthohumol, demethoxycurcumin, morellic acid, a-solanine, exhibited inhibition rates exceeding 75% against HCC827OR cell viability (Fig. 2B). Further investigation revealed that only xanthohumol demonstrated over 70% inhibitory rates on cell viability against both HCC827OR and H1975OR (Fig. 2C), we, therefore, focus on xanthohumol for further study. HCC827OR and H1975OR cells were treated with different concentrations of xanthohumol. The results revealed that the IC_50_ values of Xanth for HCC827OR and H1975OR were 4.365 μM and 3.093 μM, respectively (Fig. 2D). Therefore, concentrations of 0 μM, 1.5 μM, 3 μM, and 6 μM were selected for subsequent experiments. To further elucidate the inhibitory effect of xanthohumol on osimertinib-resistant NSCLC cells, soft agar analysis was conducted. The data suggested that a dose-dependent inhibition of colony formation ability of HCC827OR and H1975OR cells was presented (Fig. 2E). Moreover, the IF results indicated a dose-dependent suppression of p-Histone H3 Ser10 expression in both HCC827R and H1975R cells (Fig. 2F), suggesting a significant reduction in cell proliferation capability post xanthohumol treatment. Moreover, the results of the patient-derived lung cancer organoids showed that xanthohumol significantly reduced the number and volume of organoids (Supplementary Fig. 1). These findings collectively suggest that xanthohumol exhibits potent anti-tumor effects against osimertinib-resistant NSCLC cells, making it a promising candidate for overcoming osimertinib resistance.

### Xanthohumol induces endogenous apoptosis of osimertinib-resistant NSCLC cells

**Fig. 3:**
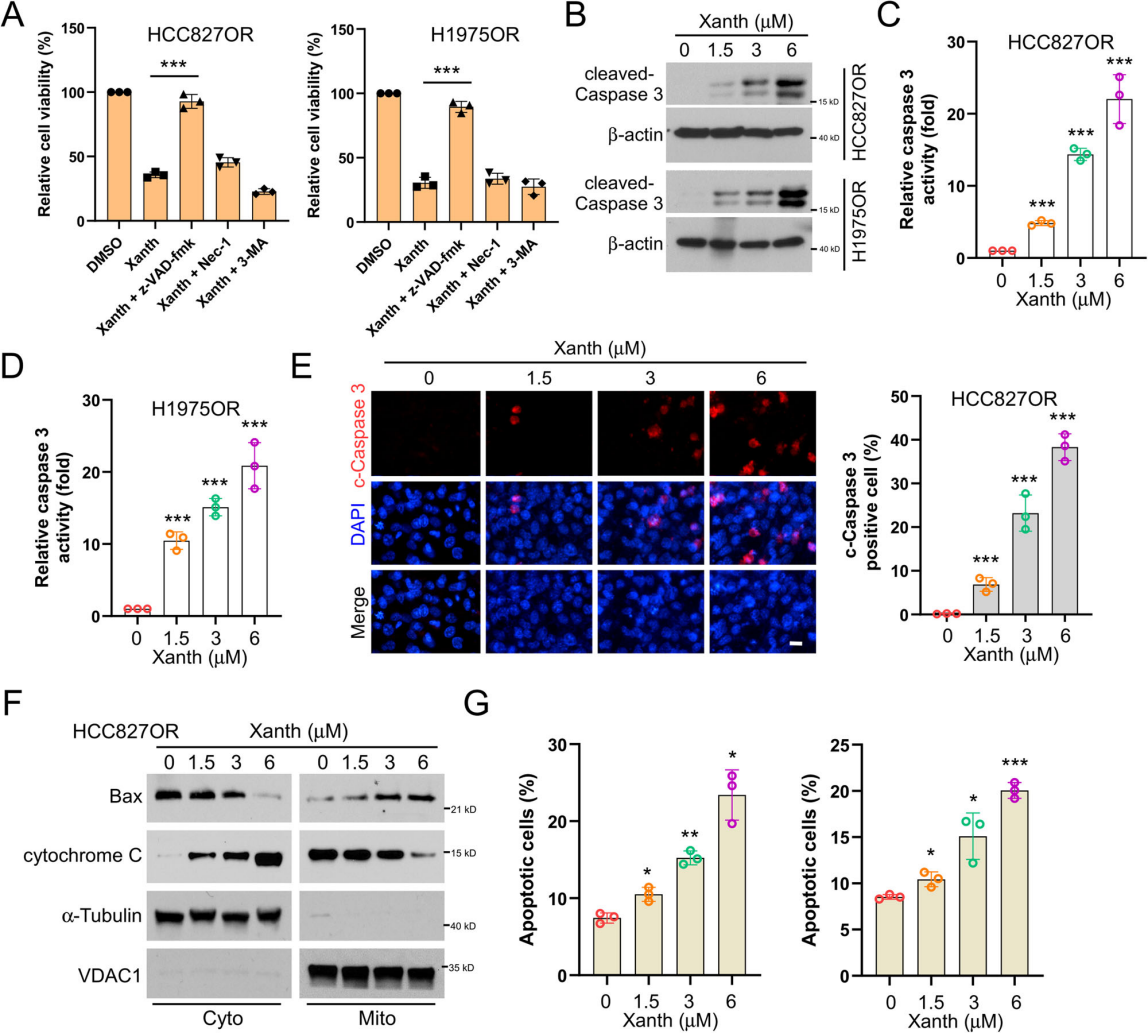
Xanthohumol promotes endogenous apoptosis in NSCLC drug-resistant cell lines. **A** HCC827OR and H1975OR cells were pretreated with z-VDA-fmk, necrostatin, or 3-MA for 4 h, then treated with xanthohumol (3 μM) for 24 h, and finally, the cell viability was analyzed by MTS assay. ***p < 0.001. The protein expression level of cleaved-Caspase 3 was detected by IB assay after 24 h of xanthohumol treatment; (**B**) caspase 3 activity was detected by caspase 3 activity assay kit for HCC827OR cells (**C**) and H1975OR cells (**D**); and c-caspase 3 positive cell number (**E**). Scale bar, 25 μM. ***p < 0.001. **F** Subcellular fractions were isolated for IB analysis After treatment of HCC827OR cells with xanthohumol for 24 h. **G** After treating HCC827OR cells with xanthohumol for 24 h, the number of apoptotic cells was analyzed by flow cytometry and statistical analysis was performed. *p < 0.05. **p < 0.01. ***p < 0.001.

Our previous findings revealed that xanthohumol significantly inhibits the cell viability and colony formation of osimertinib-resistant cells. To further elucidate how xanthohumol induces cell death in NSCLC-resistant cells, we treated cells with xanthohumol in combination with various cell death inhibitors, including apoptosis inhibitor z-VAD-fmk, the necroptosis inhibitor necrostatin-1, and the autophagy inhibitor 3-MA, respectively. The results revealed that only pre-treatment of z-VAD-fmk significantly restored cell viability (Fig. 3A). Moreover, the protein expression, the activity of cleaved-Caspase 3, and the population of cleaved-Caspase 3 positive cells in HCC827OR and H1975OR, were upregulated following xanthohumol treatment (Fig. 3B–E). Furthermore, western blot results indicate that xanthohumol promotes the release of cytochrome C from the mitochondria to the cytoplasm and facilitates the translocation of Bax to the mitochondria (Fig. 3F). Similarly, the flow cytometry data showed that following xanthohumol treatment, the proportion of apoptotic cells in HCC827OR and H1975OR cells was increased dose-dependently (Fig. 3G). These data indicate that Xanthohumol induces endogenous apoptosis of osimertinib-resistant NSCLC cells.

### Xanthohumol suppresses osimertinib-resistant NSCLC cells by inhibiting the Ets-1/c-Met axis

**Fig. 4:**
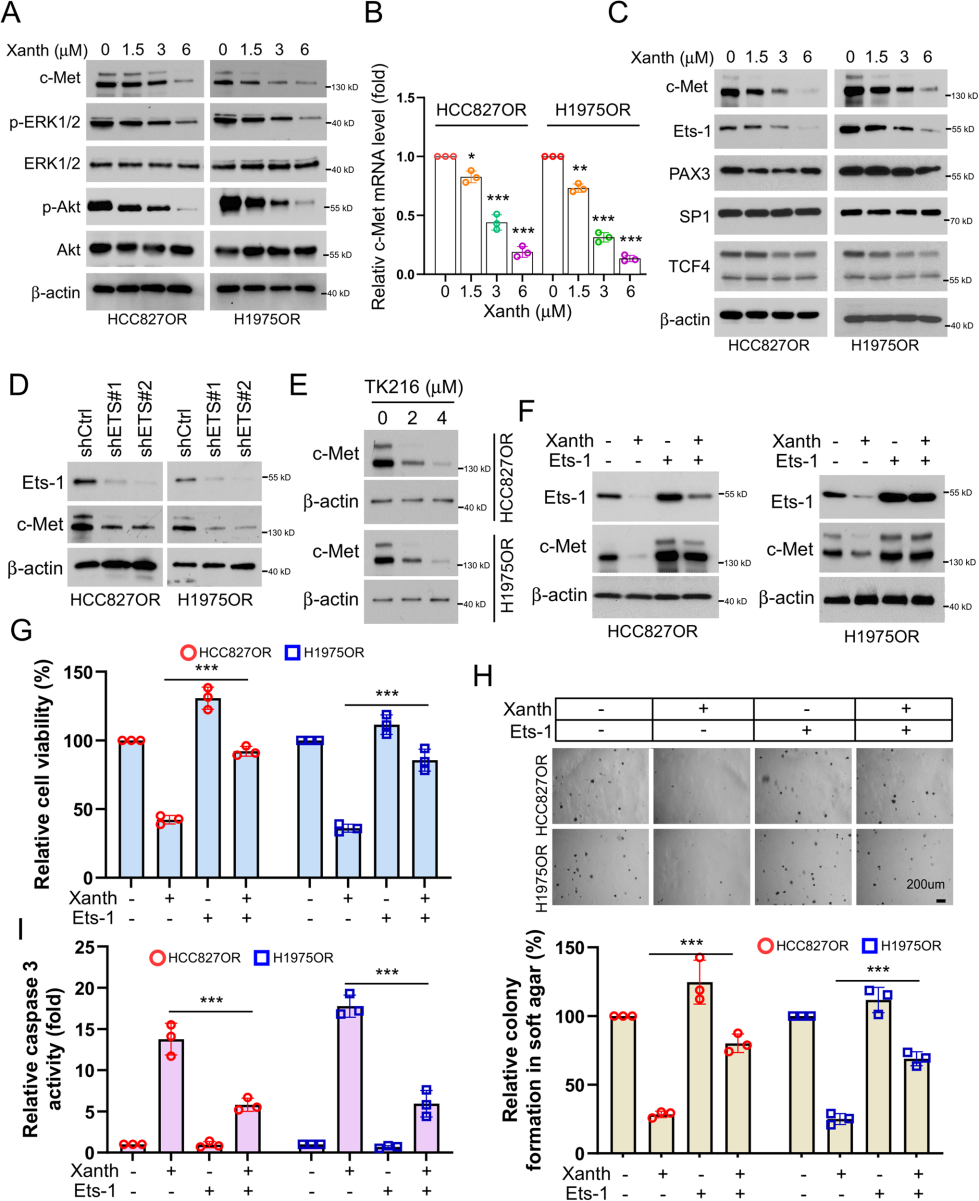
Xanthohumol suppresses osimertinib-resistant NSCLC cells by inhibiting the Ets-1/c-Met axis. After treating HCC827OR and H1975 cells with xanthohumol for 24 h, cells were collected for IB analysis (**A**, **C**) and qPCR analysis (**B**). *p < 0.05.**p < 0.01.***p < 0.001. **D** Knockdown of ETS and WB analysis of protein expression levels of Ets-1. **E** HCC827OR (top) and H1975OR cells (bottom) were treated with different concentrations of TK216 (ETS inhibitor, 0, 2, 4 μM), and the protein expression level of c-Met was analyzed by WB. HCC827OR and H1975OR cells were treated with xanthohumol, overexpressed Ets-1 or co-treated, and the protein expression levels of Ets-1 and c-Met were analyzed by WB (**F**); cell viability was analyzed by the MTS assay (**G**); colony-forming ability was detected by the soft-agar clone formation assay (**H**); and the caspase 3 activity assay kit to detect caspase 3 activity (**I**). Scale bar, 200 μm. ***p < 0.001.

To clarify whether xanthohumol affects the c-Met signaling pathway, we treated the osimertinib-resistant HCC827OR and H1975OR cells with different concentrations of xanthohumol. The immunoblotting (IB) results indicated that xanthohumol dose-dependently inhibited the expression levels of c-Met and p-ERK1/2 and p-Akt, while it had no significant effect on the total protein levels of ERK1/2 and Akt (Fig. 4A). We then examined the effect of xanthohumol on the levels of c-Met mRNA in HCC827OR and H1975OR cells and found that xanthohumol dose-dependently reduced the levels of c-Met mRNA (Fig. 4B), suggesting that xanthohumol affects its protein expression levels by regulating c-Met transcription. Previous studies have demonstrated that transcription factors, including Ets-1, PAX3, SP1, and TCF4, regulate c-Met transcription [24, 26, 36, 37]. Therefore, we further investigated the effect of xanthohumol on these transcription factors and found that xanthohumol dose-dependently downregulated the expression levels of Ets-1 protein, while it had no significant effect on the expression of PAX3, SP1, and TCF4 (Fig. 4C). Moreover, xanthohumol significantly reduced the mRNA levels of Ets-1 downstream target genes (Supplementary Fig. 2). To clarify the regulation of c-Met by Ets-1 in osimertinib-resistant NSCLC cells, we first established cell lines with stable knockdown of Ets-1 and found that silencing Ets-1 downregulated the protein levels of c-Met (Fig. 4D). Similarly, TK216, an ETS inhibitor, dose-dependently inhibited c-Met expression in HCC827OR and H1975OR cells (Fig. 4E). Previous reports indicate that TK-216 and YK-4-279 are both inhibitors of the ETS family, each with distinct advantages [38–40]. YK-4-279 is notable for its strong anti-tumor activity in preclinical models, although it may cause toxicity to normal cells and lacks long-term safety data. In contrast, TK-216 demonstrates superior efficacy and potentially reduced off-target effects compared to YK-4-279. Additionally, TK-216 is designed to specifically bind to the EWS-FLI1 fusion protein, which reduces potential interactions with other proteins and minimizes unintended side effects. However, like its counterpart, TK-216 requires a thorough clinical evaluation to fully understand its safety profile and address potential resistance. In this study, we chose TK-216 as an inhibitor of Est-1, aligning with our goal of optimizing both therapeutic efficacy and safety. Furthermore, Ets-1 overexpression restored the levels of c-Met in xanthohumol-treated HCC827OR and H1975OR cells (Fig. 4F). Additionally, Ets-1 overexpression significantly compromised the inhibitory effects of xanthohumol on the viability and clone formation ability in soft agar of HCC827OR and H1975OR cells (Fig. 4G, H). Overexpression of Ets-1 also significantly reduced caspase 3 activity in xanthohumol-treated HCC827OR and H1975OR cells (Fig. 4I), suggesting that Ets-1 overexpression significantly impaired xanthohumol-induced apoptosis of HCC827OR and H1975OR cells. These results indicate that xanthohumol inhibits the expression of c-Met by suppressing its upstream transcription regulator Ets-1.

### Xanthohumol facilitates Ets-1 ubiquitination and degradation by disturbing the interaction with USP9X

**Fig. 5:**
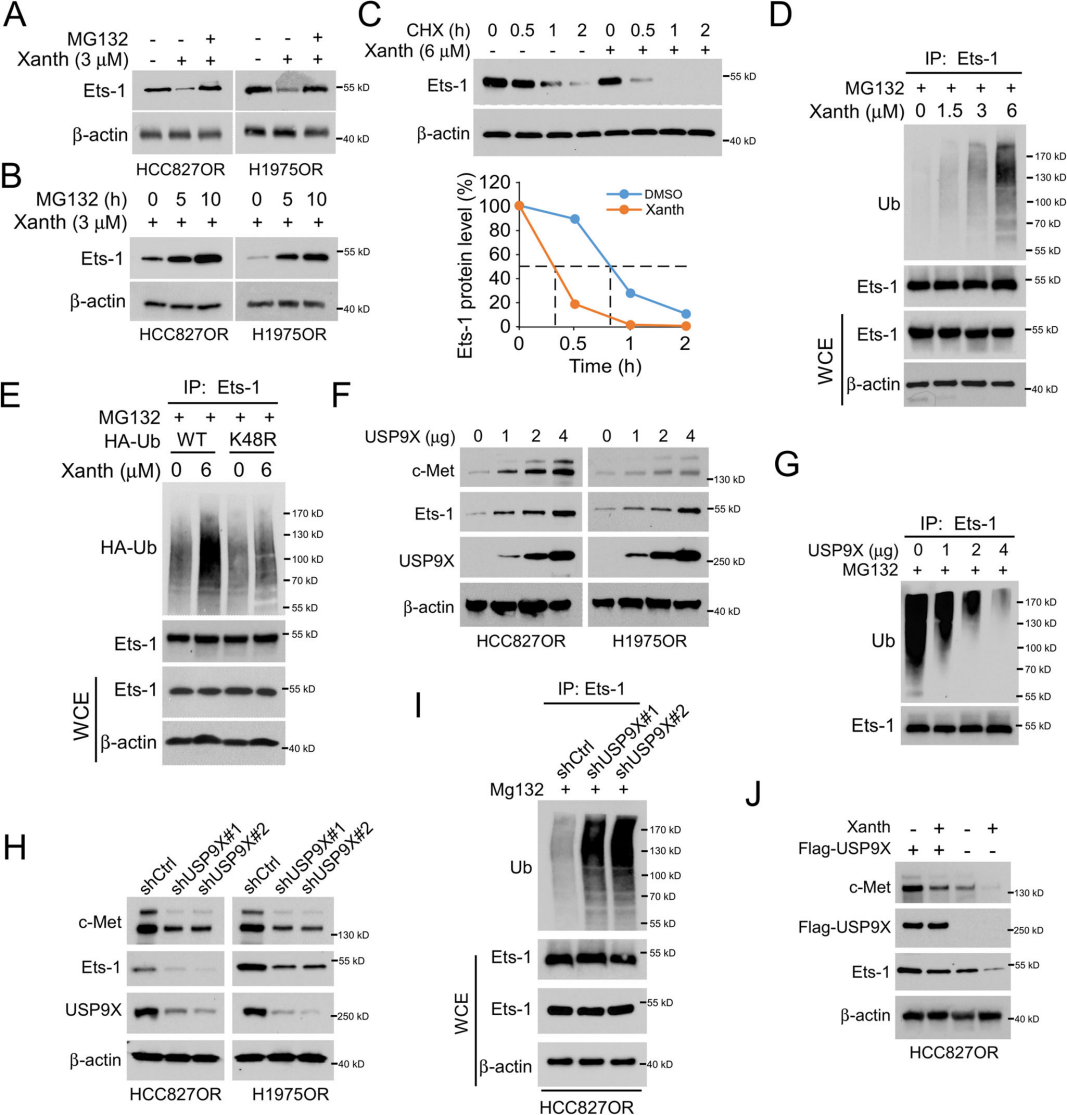
Xanthohumol promotes ubiquitination and degradation of Ets-1. **A** HCC827OR and H1975OR cells were treated with xanthohumol (3 μM) for 24 h, MG132 (10 μM) for 6 h or co-treated, and IB analysis of WCE was performed. **B** HCC827OR and H1975OR cells were treated with different concentrations of MG132 (0, 5, 10 μM) for 6 h combined with xanthohumol (3 μM) for 24 h, and IB analysis of WCE was performed. **C** HCC827OR cells were treated with/without xanthohumol (6 μM) for 24 h. Cycloheximide (CHX) was added to the medium and incubated for different times (0, 0.5, 1, 2 h), and IB analysis was performed on WCE. **D** Treatment with different concentrations of xanthohumol (0, 1.5, 3, 6 μM) for 24 h, followed by incubation with MG132 for 6 h. WCE was collected to detect the ubiquitination level of Ets-1. **E** After transfection of HA-Ub and K48R plasmids in HCC827OR cells for 24 h, respectively, treatment with or without xanthohumol (6 μM) was given for 24 h, followed by incubation with MG132 for another 6 h. WCE was collected for ubiquitination analysis. **F** IB analysis of WCE in HCC827OR and H1975OR cells transfected with different masses of USP9X plasmid. **G** USP9X plasmids of different masses were transfected in HCC827OR cells and after 24 h of transfection, MG132 was added and treated for 6 h. WCE was collected to detect the ubiquitination level of Ets-1. **H** Stable cell lines with knockdown USP9X were constructed in HCC827OR and H1975OR cells and WCE was collected for IB assay. **I** In USP9X-deficient HCC827OR cells, MG132 was added and incubated for 6 h, and Ub assay was performed to analyze the ubiquitination level of Ets-1. **J** In HCC827OR cells, xanthohumol treatment, overexpression of USP9X, or co-treatments were given, and WCE was collected for IB analysis.

To further elucidate the specific mechanism by which xanthohumol inhibits the transcription factor Ets-1, we first examined the effect of different concentrations of xanthohumol on the expression of Ets-1 mRNA in HCC827OR and H1975OR cells. The results showed that xanthohumol did not significantly affect the mRNA levels of Ets-1 (Supplementary Fig. 3A). Furthermore, MG132 treatment restored the protein levels of Ets-1 in xanthohumol-treated HCC827OR and H1975OR cells (Fig. 5A), with a noticeable increase in Ets-1 protein levels as the treatment time extended (Fig. 5B). We further investigated the effect of xanthohumol on the stability of Ets-1 protein and found that xanthohumol significantly shortened the half-life of Ets-1 in HCC827OR cells with xanthohumol treatment (Fig. 5C). These results suggest that xanthohumol could downregulate the protein levels of Ets-1 through proteasomal degradation pathway. Therefore, we examined the ubiquitination levels of Ets-1 in HCC827OR cells treated with different concentrations of xanthohumol. The results showed that Ets-1 ubiquitination levels were increased in a dose-dependent manner following xanthohumol treatment (Fig. 5D). To clarify whether xanthohumol affects Ets-1 Ub-K48 polyubiquitination chains, we transfected HA-Ub WT and K48R mutant plasmids into HCC827OR cells. The IB results indicated that HA-Ub K48R, but not WT, impaired xanthohumol-induced Ets-1 ubiquitination (Fig. 5E). Previous studies have suggested that the deubiquitinase USP9X is involved in regulating the ubiquitination levels of Ets-1 [41]. However, its effect on Ets-1 ubiquitination in NSCLC cells remains unclear. Our data showed that the expression of Ets-1 and c-Met upregulated with increasing amounts of transfected USP9X (Fig. 5F), while the ubiquitination levels of Ets-1 decreased and c-Met mRNA was increased (Fig. 5G and Supplementary Fig. 3B). To further elucidate the effect of USP9X on Ets-1 and c-Met, we established stable cell lines with USP9X knockdown and examined Ets-1 and c-Met expressions (Fig. 5H). The IB results showed that the knockdown of USP9X decreased the protein levels of Ets-1 and c-Met (Fig. 5H). Additionally, the ubiquitination of Ets-1 was increased in USP9X knockdown HCC827OR cells (Fig. 5I). To determine whether USP9X affects the inhibitory effect of xanthohumol on Ets-1 and c-Met, we transfected Flag-USP9X into HCC827OR cells. The results indicated that USP9X overexpression promoted the protein levels of Ets-1 and c-Met in xanthohumol-treated HCC827OR cells (Fig. 5J). Furthermore, USP9X overexpression significantly restored the viability and clonogenic capacity in soft agar of xanthohumol-treated HCC827OR and H1975OR cells (Supplementary Fig. 3C, D). Similarly, USP9X overexpression significantly reduced caspase 3 activity in xanthohumol-treated HCC827OR and H1975OR cells (Supplementary Fig. 3E), suggesting that USP9X overexpression reduces the apoptosis of xanthohumol-treated HCC827OR and H1975OR cells. In the GEO database, the expression levels of MET, ETS-1, and USP9X in osimertinib-resistant lung cancer cells, as observed in the datasets GSE202859 and GSE236654, were significantly higher than those found in osimertinib-sensitive lung cancer cells (Supplementary Fig. 4A, B). Likewise, in GSE253742, MET, ETS-1, and USP9X expression levels in lung cancer tissues after osimertinib treatment were elevated compared to untreated lung cancer tissues, supporting our research findings (Supplementary Fig. 4C). The data above indicates that xanthohumol promotes Ub-K48-mediated polyubiquitination and degradation of Ets-1.

### Xanthohumol destabilizes Ets-1 in a Thr38 phosphorylation-dependent manner

**Fig. 6:**
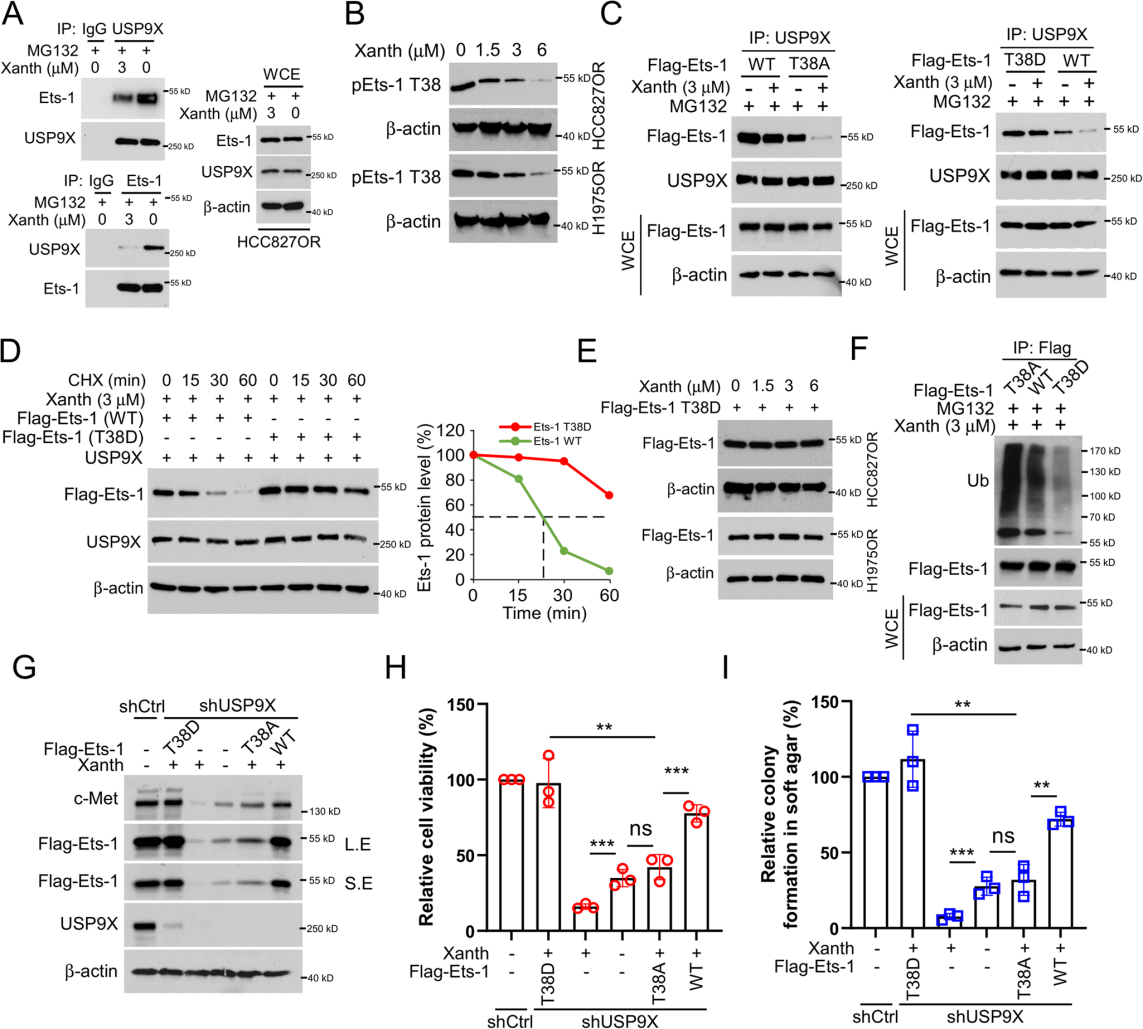
Xanthohumol destabilizes Ets-1 in a Thr38 phosphorylation-dependent manner. **A** HCC827OR cells, treated with/without xanthohumol for 24 h, were subsequently incubated with the addition of MG132 for an additional 6 h. Immunoprecipitation (IP) assays were performed to detect the interaction of USP9X and Est-1. **B** HCC827OR and H1975OR cells were treated with different concentrations of xanthohumol and subjected to IB analyses for WCE. **C** Flag-Est-1 WT, T38A and T38D were transfected into HCC827OR cells for 24 h, xanthohumol (3 μM) treatment was performed for 24 h, MG132 was added to the medium and incubated for 6 h. IB analysis of WCE was performed. **D** The corresponding plasmids were transfected into HCC827OR cells for 24 h. The cells were treated with 3 μM xanthohumol for 24 h. Then, the cells were incubated with CHX (20 μg/ml) at different times, and WCE was collected for IB analysis. **E** In HCC827OR and H1975OR cells, the cells were transfected with Flag-Ets-1 T38D plasmid for 24 h and then treated with different concentrations of xanthohumol for 24 h. WCE was collected for IB analysis. **F** Flag Ets-1 WT, T38A, or T38D were transfected into HCC827OR cells for 24 h. The cells were given xanthohumol treatment for 24 h and MG132 treatment for 6 h. The ubiquitination level of Ets-1 was analyzed by IB. IB analysis of WCE in HCC827OR cells transfected/not transfected with Flag Ets-1 WT, T38A, or T38D plasmids and cells treated with/without xanthohumol (**G**); cell viability analyzed by the MTS assay (**H**); and colony-forming ability detected by the soft-agar clone formation assay (**I**). Scale bar, 200 μm. **p < 0.01. ***p < 0.001.

We examined the interaction between USP9X and Ets-1 in the presence of Xanth. The data indicated a mutual binding interaction between USP9X and Ets-1 within HCC827OR cells, which was attenuated by xanthohumol (Fig. 6A). Furthermore, treatment of HCC827OR and H1975OR cells with different concentrations of xanthohumol resulted in a concentration-dependent decrease in phosphorylation of Ets-1 on Thr38 (Fig. 6B). Previous studies suggested a correlation between protein phosphorylation and stability [42]. However, the impact of Ets-1 phosphorylation levels on its stability remained unclear. Hence, we constructed Flag-Ets-1 T38A (mimicking phosphorylation-inactive state) and T38D (mimicking constitutively phosphorylated state) expression plasmids. Flag-Ets-1 WT, T38A, or T38D plasmids were transfected into HCC827OR cells and subjected to xanthohumol treatment. The Co-IP and IB results revealed that xanthohumol exhibited a more substantial inhibitory effect on the binding of USP9X to Ets-1 T38A. In contrast, Flag-Ets-1 T38D mutant markedly impaired this efficacy (Fig. 6C). Cycloheximide assay was next performed. The results showed that compared to Flag-Ets-1 WT, Flag-Ets-1 T38D overexpression prolonged the half-life of Ets-1 (Fig. 6D). Additionally, overexpression of Flag-Ets-1 T38D restored the expression levels of Ets-1 in HCC827OR and H1975OR cells after treatment with different concentrations of xanthohumol (Fig. 6E). Moreover, xanthohumol promoted stronger ubiquitination of the T38A mutant than that of the WT and T38D (Fig. 6F), indicating that Thr38 phosphorylation stabilized Ets-1 protein. To elucidate the underlying mechanism, we examined the ubiquitination and protein expression levels of Ets-1 T38 with different phosphorylation statuses after xanthohumol treatment. The IB results showed that transfection with Flag-Ets-1 T38D substantially restored Ets-1 and c-Met expression even in the presence of xanthohumol (Fig. 6F, G). Furthermore, Flag-Ets-1 T38D significantly restored cell viability and clone-forming ability in soft agar of xanthohumol-treated HCC827OR and H1975OR cells (Fig. 6H, I). These results suggest that the phosphorylation of Est-1 at T38 plays a crucial role in regulating its stability.

### Xanthohumol inhibits the in vivo tumor growth of osimertinib-resistant NSCLC cells

**Fig. 7:**
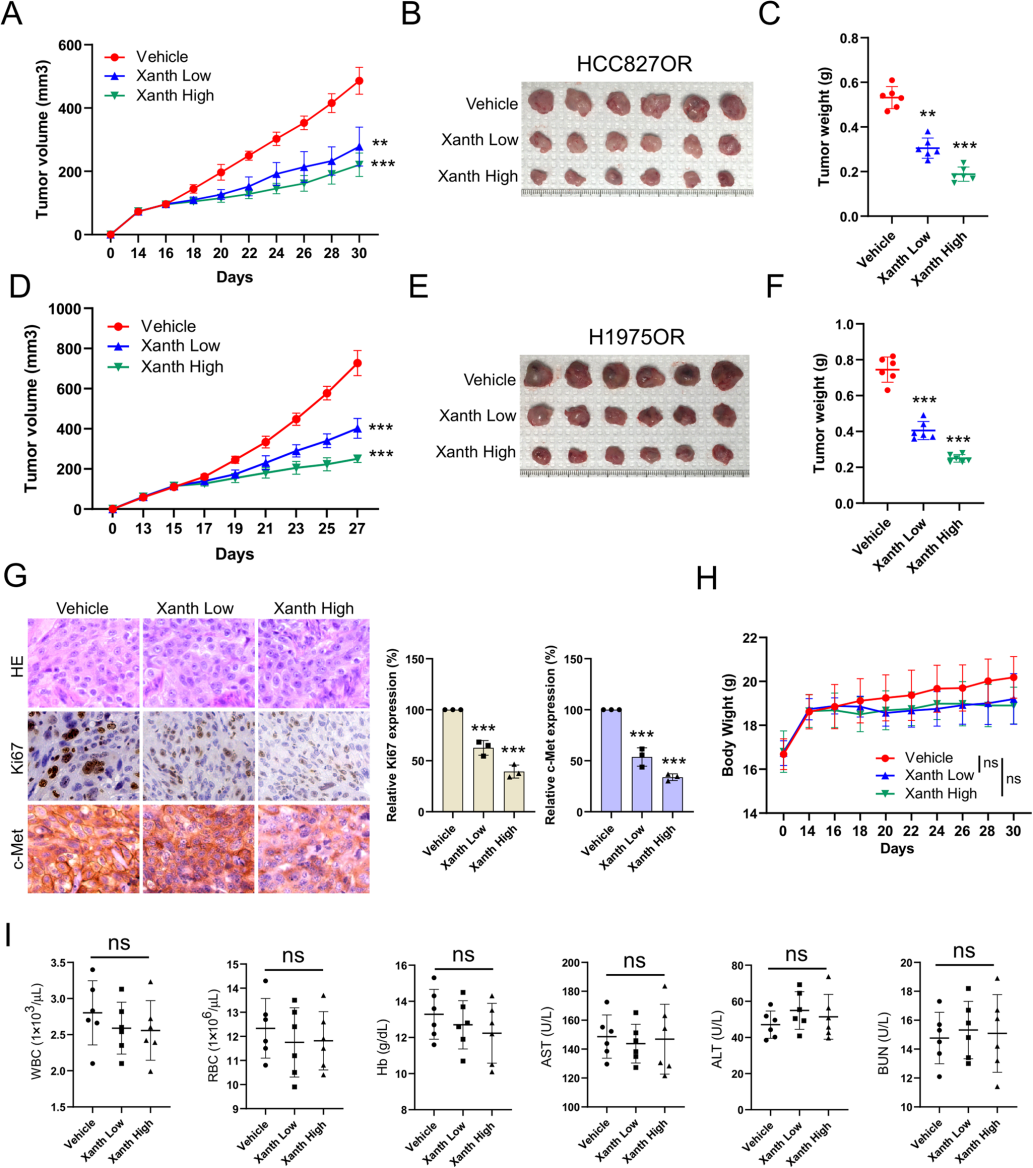
Xanthohumol inhibits in vivo tumor growth in osimertinib-resistant cells. The xenograft tumor model analyzed the effect of xanthohumol administration concentration (Low: 10 mg/kg/2 days; High: 30 mg/kg/2 days) on HCC827OR tumor volume (**A**), tumor mass (**B**) and tumor weight (**C**). **p < 0.01.***p < 0.001. Xenograft tumor model analyzing the effect of xanthohumol administration concentration on H1975OR tumor volume (**D**), tumor mass (**E**) and tumor weight (**F**). **p < 0.01.***p < 0.001. **G** IHC method to analyze the effect of xanthohumol administration concentration on the expression levels of Ki67 and c-Met in HCC827OR tumor tissues. IHC images are shown on the left, and statistical analysis is shown on the right. Scale bar, 25 μm. ***p < 0.001. **H** Changes in body weight of HCC827OR transplanted tumor-bearing mice treated with control or different concentrations of xanthohumol administration. ns indicates no statistical significance. **I** WBC, RBC, Hb, AST, ALT, and BUN in blood samples of HCC827OR transplanted tumor-bearing mice treated with control or different concentrations of xanthohumol administration were analyzed. ns indicates no statistical significance.

To further investigate the effect of xanthohumol on NSCLC osimertinib-resistant cell lines in vivo, we established xenograft mouse models using HCC827OR and H1975OR cells. The results demonstrated that xanthohumol could inhibit the growth of osimertinib-resistant cells in vivo dose-dependently. A smaller tumor volume, tumor size, and tumor weight were observed in the high-dose xanthohumol treatment group (Fig. 7A–F). Immunohistochemistry (IHC) staining showed that the expression levels of Ki67 and c-Met decreased after xanthohumol treatment, with a more significant efficacy observed at high doses (Fig. 7G). Additionally, there was no significant change in body weight in nude mice with or without xanthohumol treatment (Fig. 7H). Analysis of blood samples showed no significant impact of xanthohumol on white blood cells (WBC), red blood cells (RBC), hemoglobin (Hb), aspartate aminotransferase (AST), alanine aminotransferase (ALT), and blood urea nitrogen (BUN) levels (Fig. 7I). In addition, immunohistochemical analyses of the heart, liver, spleen, lung and kidney of tumor-bearing mice showed that xanthohumol did not affect the essential functions of internal organs (Supplementary Fig. 5). These results indicate that xanthohumol treatment significantly inhibits tumor growth and is well tolerated in vivo.

### Xanthohumol overcomes osimertinib resistance for NSCLC cells

**Fig. 8:**
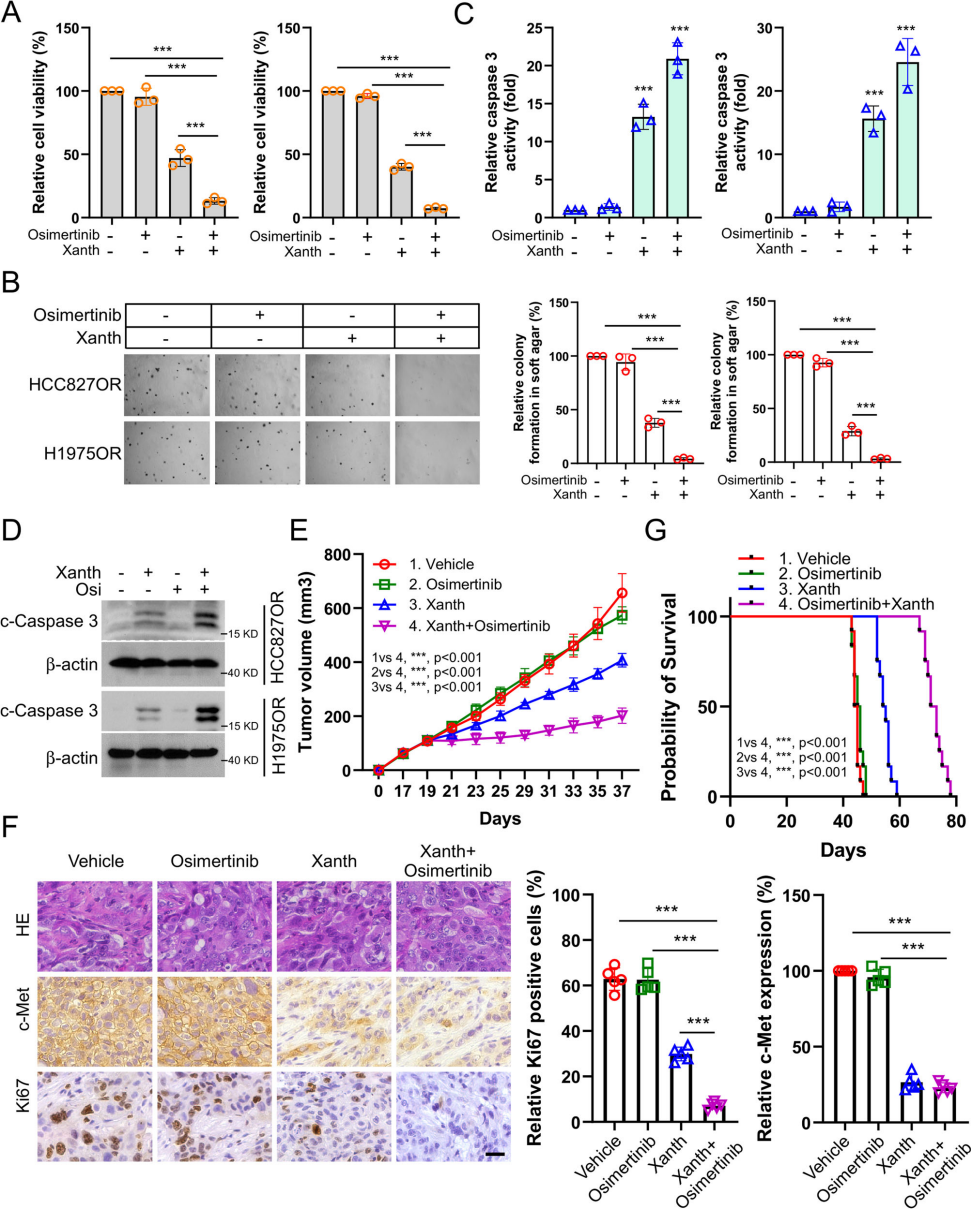
Xanthohumol overcomes osimertinib resistance in NSCLC cells in vitro and in vivo. Administration of xanthohumol, osimertinib, or combination treatment in HCC827OR and H1975OR cells. Cell viability was analyzed by MTS assay (**A**); colony forming ability was analyzed by soft agar clone formation assay (**B**); caspase 3 activity was detected by caspase 3 activity assay kit (**C**); IB method to analyze c-Caspase 3 protein expression level (D). Scale bar, 200 μm. ***p < 0.001. A xenograft tumor model was constructed using HCC827OR cells, and mice were given xanthohumol, osimertinib, or co-treatment to examine the anti-tumor effects. The volume was recorded (**E**), the expression of Ki67 and c-Met in tumor tissues was examined (**F**), and the survival time of mice was recorded (**G**). Scale bar, 25 μm. ***p < 0.001.

To further determine whether xanthohumol could restore the sensitivity of NSCLC Osimertinib-resistant cells to Osimertinib. We treated osimertinib-resistant cell lines with xanthohumol and showed that xanthohumol, but not osimertinib, reduced cell viability (Fig. 8A) and cell colony-forming ability (Fig. 8B) and significantly increased the activity level of caspase 3 (Fig. 8C). In addition, immunoblotting results showed a significant increase in the protein expression level of cleaved-Caspase 3 (Fig. 8D), and all of these effects were further enhanced upon the combination with osimertinib treatment. In a mouse transplantation tumor model, xanthohumol, but not osimertinib, was observed to reduce tumor growth and volume, and the anti-tumor effect was further enhanced by co-administration (Fig. 8E). IHC results showed that the co-administration group significantly reduced the expression of Ki67 and c-Met (Fig. 8F). In addition, as shown in Fig. 8G, the combination group significantly increased the survival rate of tumor-bearing mice. These results suggest that xanthohumol treatment overcomes osimertinib resistance in NSCLC cells.

## Discussion

Although the clinical anti-tumor efficacy of osimertinib in NSCLC patients with EGFR-activation mutations has been proved, there is variability in the duration of response, with patients eventually developing acquired resistance [43]. Recent studies have highlighted the complex interplay between oncogenic drivers and tumor suppressor genes as crucial factors influencing treatment response in NSCLC [44, 45]. For example, recent findings have expanded our understanding of osimertinib resistance mechanisms beyond previously known EGFR mutations, such as the EGFR C797S mutation. New research has identified additional resistance mechanisms, including MET amplification and aberrant activation of bypass pathways, such as HER2 and IGF1R, and novel oncogene fusions involving RET, BRAF, and ALK [46–48]. Additionally, the dysregulation of proteins such as ST3GAL4, ZNF263, MUC1-C, DUSP1, and PTEN impacts the sensitivity of NSCLC cells to osimertinib. Moreover, emerging evidence suggests that alterations in the PI3K/AKT and RAS/MAPK/ERK signaling pathways are critical in mediating resistance, indicating a need for a broader exploration of these signaling networks [49–52]. However, no promising predictive biomarkers are currently available for clinical improvement of osimertinib resistance. Therefore, further research is needed to investigate osimertinib-resistant biomarkers and their potential mechanisms of action to develop effective treatment strategies for NSCLC patients. Herein, we found that c-Met expression is significantly upregulated in osimertinib-resistant NSCLC cells, and knockdown of c-Met resulted in a significant decrease in tumorigenicity both in vitro and in vivo, with a significant extension in the survival period of tumor-bearing mice with c-Met downregulation. Overall, we identified biomarkers associated with osimertinib resistance, such as c-Met, which can enhance our understanding of the mechanisms underlying osimertinib resistance and contribute to the development of more effective strategies for overcoming resistance in NSCLC patients.

It is reported that the overexpression of c-Met occurs in about 20–25% of patients with NSCLC and persistently stimulates PIK3CA, MAPK, and STAT pathways to bypass EGFR, thereby inducing the malignant progression of the tumor [53]. In addition to genomic MET amplification and Exon 14 mutations being associated with c-Met overexpression, several transcription factors like hypoxia-inducible factor 1α, FOXC2, PAX3, SP1, TCF4, and Ets-1 are involved in the regulation of c-Met expression and activation [24, 36, 37, 54, 55]. Moreover, c-MET expression is also post-transcriptionally regulated by TP53-induced glycolysis and apoptosis regulator, c-Cbl and CHIP [56–58]. However, the involvement of transcription factors in regulating c-Met expression levels in acquired resistance to osimertinib in NSCLC remains unclear. Our study suggests that the upregulation of c-Met expression in osimertinib-resistant NSCLC cells is attributed to the transcription factor Ets-1, rather than PAX3, SP1, or TCF4. These findings showed that Ets-1 could serve as a potential therapeutic target in osimertinib-resistant NSCLC patients, but further studies are needed to confirm this.

Ets-1 is a critical transcription factor involved in various cellular processes, including cell apoptosis, differentiation, proliferation, and angiogenesis [59–61]. Ets-1 dysregulation leads to an imbalance in the expression of oncogenes and tumor suppressor genes, thereby promoting tumor progression [62, 63]. The expression of the Ets-1 gene is regulated at the transcriptional level, with its promoter sequence containing various potential binding sites for proteins such as Ets, SP1, AP-1, and AP-2 [64]. These binding sites are present in many promoters and can mediate the response of promoters to the Ras/Raf/MEK/ERK1/2 pathway through Ets-1 and other ETS proteins [19, 65]. In addition, some growth factors such as HGF and VEGF can activate the Ras/Raf/MEK/ERK1/2 and PI3K/Akt pathways, thereby upregulating Ets-1 mRNA levels and protein activity [66–68]. Other factors such as HIF-1, HRE, ARE, Nrf2, and TGFβ also have effects on upregulating Ets-1 mRNA levels or protein activity, while NRE1, NRE2, and p53 WT negatively regulate Ets-1 transcription [9, 65, 69]. In addition to transcriptional regulation, Ets-1 can be regulated during translation and post-translational modification. For example, some microRNAs, such as miR-193b, and miR-129-5p, can downregulate Ets-1 expression and reduce the migration and invasion capabilities of tumor cells [70, 71]. Activation of the Ras/Raf/MEK/ERK1/2 pathway can phosphorylate the Ets-1 protein at the Thr38 site [72]. Growth factor receptors such as HGF and RAS responding to NO signals induce S-nitrosylation of RAS-dependent Ets-1 Thr38 phosphorylation, and connective tissue growth factor induces ERK1/2-dependent Ets-1 Thr38 phosphorylation [19, 73]. However, the functional connection between the Ras/Raf/MEK/ERK1/2 pathway and Ets-1 is bidirectional, as Ets-1 can upregulate the expression of this signaling cascade’s components and enhance its activity [19, 74]. For example, in melanoma cells, Ets-1 cooperates with PAX3 to increase the transcription of the c-Met gene [24]. Our research indicates that xanthohumol can inhibit the phosphorylation levels of ERK1/2, but it does not affect the expression levels of Est1 mRNA. Instead, it promotes the degradation of Est1 through the ubiquitin-proteasome system. We explored how xanthohumol affects Ets-1 phosphorylation and degradation and provided insights into potential therapeutic approaches for cancers driven by Ets-1 dysregulation. This understanding may ultimately contribute to the development of targeted treatments aimed at modulating Ets-1 activity to inhibit tumor progression.

The turnover of the Ets-1 protein is regulated by ubiquitinases and deubiquitinases, such as COP1, SYVN1, HUWE1, and USP9X [41, 75–77]. In this study, we found that the deubiquitinase USP9X interacts with Ets-1 and regulates Ets-1 ubiquitination levels in osimertinib-resistant NSCLC cells. Overexpression of USP9X reverses the pro-apoptotic effect of xanthohumol on osimertinib-resistant cell lines HCC827OR and H1975OR. Overexpression of Flag-Ets-1 T38A augments xanthohumol-induced downregulation of Ets-1, while overexpression of Flag-Ets-1 T38D inhibits xanthohumol-induced downregulation of Ets-1. Moreover, overexpression of Flag-Ets-1 T38D significantly prolongs the half-life of Ets-1. Further investigation revealed that compared to overexpression of Flag-Ets-1 WT, overexpression of Flag-Ets-1 T38A potentiates xanthohumol-induced ubiquitination of Ets-1, while overexpression of Flag-Ets-1 T38D mitigates xanthohumol-induced ubiquitination of Ets-1. Meanwhile, overexpression of Flag-Ets-1 T38D restores the expression of c-Met in xanthohumol-treated HCC827OR cells and restores cell viability, while overexpression of Flag-Ets-1 T38A further downregulates the expression of c-Met in xanthohumol-treated HCC827OR cells and promotes apoptosis. These results indicate that phosphorylation of the T38 site of Ets-1 regulates its stability and is associated with osimertinib resistance. Overall, the study provides valuable insights for sensitizing resistant NSCLC cells to osimertinib, potentially improving treatment outcomes for patients with drug-resistant cancer.

Natural products are considered highly promising anti-tumor drugs due to their distinct anti-tumor effects and ability to overcome the resistance of tumor cells to radiotherapy and chemotherapy [33, 78]. For example, curcumin inhibits NSCLC cells by targeting the PI3K/Akt/mTOR axis [79]. Gastrodin overcomes cisplatin resistance in oral squamous cell carcinoma cells by inhibiting the Akt/HK2 axis [80]. Gastrodin inhibits lung cancer cells resistant to pemetrexed by promoting survivin degradation through the inhibition of the Akt/WEE1/CDK1 signaling pathway [81]. The in vitro and in vivo data presented here demonstrate that xanthohumol can target the Ets-1/c-Met signaling pathway to inhibit the growth of osimertinib-resistant NSCLC cells by interfering with the binding of USP9X to Ets-1. Additionally, piceatannol exhibits relatively non-toxic properties in vivo. Of note, research shows xanthohumol not only sensitizes oral squamous cell carcinoma to radiotherapy by inhibiting the Akt-Wee1-CDK1 signaling axis but also induces apoptosis in various cancers, including breast cancer, prostate cancer, melanoma, and glioma, by suppressing molecular pathways related to EGFR, MDR1, STAT3, HK2, NF-κB, ERK1/2, and DNA topoisomerase 1, thereby achieving anti-tumor effects [32, 82–84]. Moreover, some of these molecules overlap with those involved in the osimertinib resistance mechanisms in NSCLC mentioned above, suggesting that xanthohumol might exert its sensitizing effects on osimertinib through multiple pathways. Overall, this study explored natural products as potential anti-cancer agents, particularly focusing on xanthohumol and its effects on osimertinib-resistant NSCLC cells, which highlighted how xanthohumol targets the Ets-1/c-Met signaling pathway and interferes with USP9X binding to Ets-1, thereby inhibiting tumor growth. Additionally, this work underscores the potential of xanthohumol as a promising anti-cancer compound and further research is needed to understand its mechanisms and therapeutic applications, including in vivo and clinical studies to assess its efficacy and safety in cancer patients.

## Conclusions

In summary, this study demonstrates that xanthohumol directly attenuates Ets-1 phosphorylation and its interaction with USP9X, contributing to Ets-1 degradation and the inhibition of Ets-1/c-Met axis, thereby inducing apoptosis activation in osimertinib-resistatant NSCLC cells. Additionally, we found that the combined application of xanthohumol and osimertinib could enhance the sensitivity of NSCLC cells to osimertinib in vivo and in vitro. Therefore, targeting or inactivating the Ets-1/c-Met axis could be a promising strategy for the clinical management of NSCLC.

## Materials and methods

### Cell lines and cell culture

The NSCLC cell lines HCC827 and H1975 involved in this study were purchased from the American Typical Culture Collection (ATCC, Manassas, VA). Cells were cultured in a 37 °C incubator with 5% CO_2_ and a certain humidity level. The medium used for both cell lines was RPMI-1640 medium (containing 10% FBS and 1% penicillin-streptomycin mixture). The osimertinib-resistant HCC827OR cells with c-Met overexpression were a kind gift from Dr. Zigang Dong [85], and H1975OR cells with c-Met overexpression were generated in our lab.

### Reagents & antibodies

Inhibitors involved in the study, including MG132, cycloheximide (CHX), Necrostatin-1 (Nec-1), z-VAD-FMK and 3-MA were purchased from Selleck Chemicals (Houston, TX). Antibodies against c-Met (REF.8198; IB: 1:1000; IHC: 1:200), cleaved-caspase 3 (REF.9664; IB: 1:1000; IHC: 1: 2000), cytochrome C (REF.11940; IB: 1:1000), Bax (REF.14796; IB: 1:1000), α-Tubulin (REF.2125; IB: 1:5000), VDAC1 (REF.4866; IB: 1: 3000), β-actin (REF.3700; IB: 1:1000), α-Tubulin (REF.2125; IB: 1:5000), ERK1/2 (REF.9102; IB: 1:2000), p-ERK1/2 (REF.4370; IB: 1:1000), p-Akt (REF.4060; IB: 1:1000), Akt (REF.4691; IB: 1:2000), Ets-1 (REF.14069; IB: 2000), PAX3 (REF.12412; IB: 1:1000), SP1 (REF.5931; IB:1:1000), TCF4 (REF.2569; IB: 1:1000), USP9X (REF.5751; IB: 1:1000), Flag-tag (REF.8146; IB: 1:1000), and Ub (REF.3936; IB: 1:1000) were obtained from Cell Signaling Technology, Inc. (Beverly, MA). Antibody against Ets-1 T38 (REF.44-1104G; IB: 1:1000) was purchased from Thermo Fisher. Antibody against Ki67 (REF.ab15580; IHC: 1:2000) is a product of Abcam (Cambridge, UK).

### MTS assay

The MTS assay was performed as described previously [86]. Cells were inoculated into 96-well plates at a density of 3 × 10^3^ and incubated at 37 °C for 1 day before being treated according to the experimental design. Subsequently, MTS reagent (REF.G3581, Promega, Madison, WI) was added to the 96-well plates, and the incubation was continued at 37 °C, protected from light, for 1–2 h. Finally, the absorbance value was measured at 490 nm using an enzyme marker.

### Soft agar assay

The soft agar assay was performed as described previously [87], cells were inoculated into 6-well plates containing Eagle’s basal medium at a density of 8 × 10^3^ cells/well. Subsequently, incubation was continued for 2 weeks in a constant temperature incubator at 37 °C, during which colonies were observed. Finally, they were counted by light microscopy.

### Protein preparation and western blotting (WB) assay

Process NSCLC cells according to experimental design, collect them, and add pre-cooled RIPA lysis buffer to obtain whole cell lysate (WCE). Measure protein concentration using Thermo Fisher Scientific’s BCA protein concentration kit (REF.22328) and prepare the protein loading solution. The western blotting was performed as described previously [88]. Separate equal protein amounts on an SDS-PAGE gel, transfer to a methanol-immersed PVDF membrane, and incubate the membrane in 5% skim milk for 1 h. After blocking, remove the solution, add the primary antibody, and incubate overnight at 4 °C. The next day, incubate with the secondary antibody for 1 h. Finally, the exposure was performed using a chemiluminescence imager.

### Immunohistochemical (IHC)

Deparaffinize tissue sections with xylene and ethanol. Perform antigen retrieval using a 10 mM sodium citrate solution. Wash sections three times with distilled water and incubate for 10 min at room temperature in 3% hydrogen peroxide to quench endogenous peroxidase. Block non-specific binding with goat serum, then incubate overnight at 4 °C with the primary antibody. The next day, wash with PBS and incubate with the secondary antibody for 45 min. Add DAB solution, counterstain with hematoxylin, and observe positive staining under a microscope.

### Immunofluorescence (IF)

After treating cells per experimental protocols, remove the medium and wash it three times with PBS. Fix cells with 4% paraformaldehyde for 10 min, then permeabilize with 0.3% Triton X-100 for 15–20 min. Wash cells three times with PBS and block with bovine serum albumin. Incubate with the primary antibody at 4 °C overnight. The next day, recover the primary antibody, wash it with PBS, and incubate it with the fluorescent secondary antibody for 30 min. Stain nuclei with DAPI and observe positive cells using fluorescence microscopy.

### Co-immunoprecipitation (Co-IP) assays

Conduct the Co-IP assay according to the previous experimental protocol [86]. After treatment, collect cell precipitates and add IP lysis buffer (REF.87788, Thermo Scientific) to prepare cell lysates. Subsequently, 1 μg of the corresponding antibody and pre-washed protein A agarose beads were added to the cell lysates and incubated at 4 °C for 24 h. Finally, protein-protein interactions were measured through immunoblotting analysis.

### Ubiquitination (Ub)analysis

The NSCLC cells were collected and lysed by 1% SDS containing RIPA lysate, followed by sonication and heating in a metal bath at 95 °C for 15 min. Cell lysates were centrifuged for 10–15 min (16,000 × *g*), and the supernatant was subjected to protein concentration determination. The corresponding volume of supernatant protein was added to 750 μl of RIPA buffer containing 0.1% SDS, followed by the appropriate antibody and agarose beads, and incubated at 4 °C overnight. The upsampling buffer was prepared the next day according to the previous protocol [89].

### Cycloheximide (CHX) assay

NSCLC cells were treated with CHX (20 μg/ml) after 24 h of Xanth treatment. Subsequently, cell lysates were collected at 0, 0.5, 1, and 2 h and subjected to WB to detect changes in the half-life of Est-1.

### qRT-PCR assay

Inoculate cells into 6-well plates at 6 × 10^5^ density. When cells reach 80–90% confluence, discard the supernatant, wash twice with PBS, and add 500 μl of Trizol reagent to lyse the cells. Extract RNA using chloroform, isopropanol, and 75% ethanol, then measure RNA concentration. Take 1000 ng of total RNA for reverse transcription using the RevertAid First Strand cDNA Synthesis Kit (REF.K16215, Thermo Fisher Scientific). Perform qRT-PCR according to the qPCR premix instructions (REF.RK21203, ABclonal).

### In vivo tumor growth

The in vivo models were conducted by injecting HCC827 shCtrl and shc-Met stable cells into the right flank of 6-week-old thymus-free nude mice (*n* = 5). Tumor volume was recorded (every 2 days) and calculated based on the formula (length × width^2^/2).

When the tumor volume reached about 1000 mm^3^, the mice were euthanized, and the tumor weight was measured, and tumor tissues were fixed in formaldehyde for immunohistochemical analysis. To investigate whether xanthohumol inhibits in vivo tumor growth in Osimertinib-resistant NSCLC cells, HCC827OR and H1975OR cells were injected into nude mice (*n* = 6) to construct a xenograft tumor model, and when the tumor volume reached 100 mm^3^, the mice were randomly divided into three groups: control, low xanthohumol (10 mg/kg/2 days) and high xanthohumol groups (30 mg/kg/2 days). When the tumors reached about 1000 mm^3^, the mice were euthanized, and the tumor tissues were fixed in formaldehyde for subsequent immunohistochemical analyses. To further demonstrate that xanthohumol overcame osimertinib resistance, HCC827OR and H1975OR cells were injected into nude mice (*n* = 5). They were randomly divided into four groups when the tumor volume reached 100 mm^3^: control, osimertinib administration (5 mg/kg/day), xanthohumol administration (10 mg/kg/2 days), and co-administration groups (xanthohumol:10 mg/kg/2day; Osimertinib:5 mg/kg/2 days). Mice were euthanized when the tumor volume reached 1000 mm^3^. Tumor tissues were subjected to immunohistochemical analysis.

### Statistical analysis

This study utilized GraphPad Prism software for statistical analysis of the data. Each experiment included at least three independent replicates. Student’s *t*-test and one-way analysis of variance (ANOVA) were employed to compare different groups. Survival analysis was conducted using the Log-rank test.

## Supplementary information

full gel

Supplementary Figures

Supplementary materials Table S1

## Data Availability

All data generated or analysed during this study are included in this published article (and its supplementary information files).
