# Supplementary material for: Xanthohumol overcomes osimertinib resistance via governing ubiquitination-modulated Ets-1 turnover
Source: Cell Death Discov. 2024 Oct 28;10:454. doi: 10.1038/s41420-024-02220-y (PMC11519634; doi:10.1038/s41420-024-02220-y)

Full gel for Figure 1

C

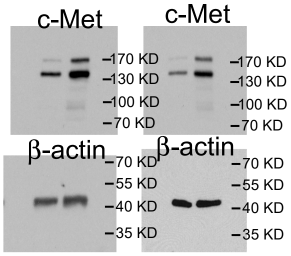

D

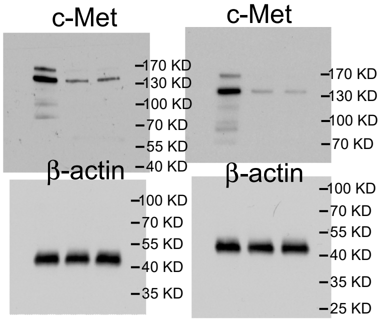

Full gel for Figure 3

B

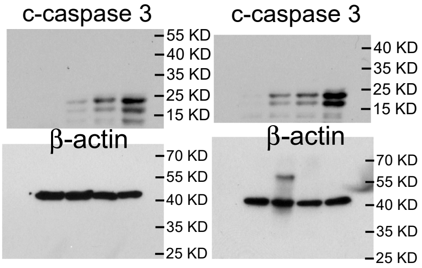

F

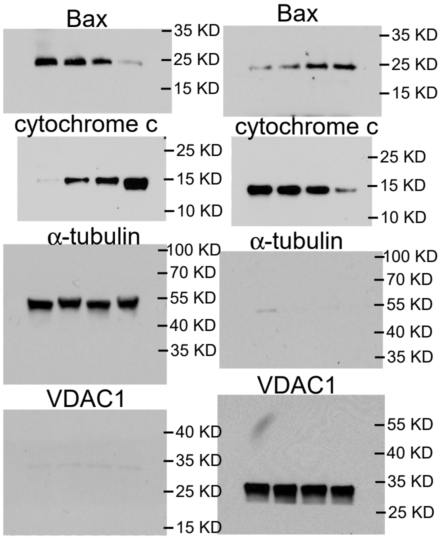

Full gel for Figure 4

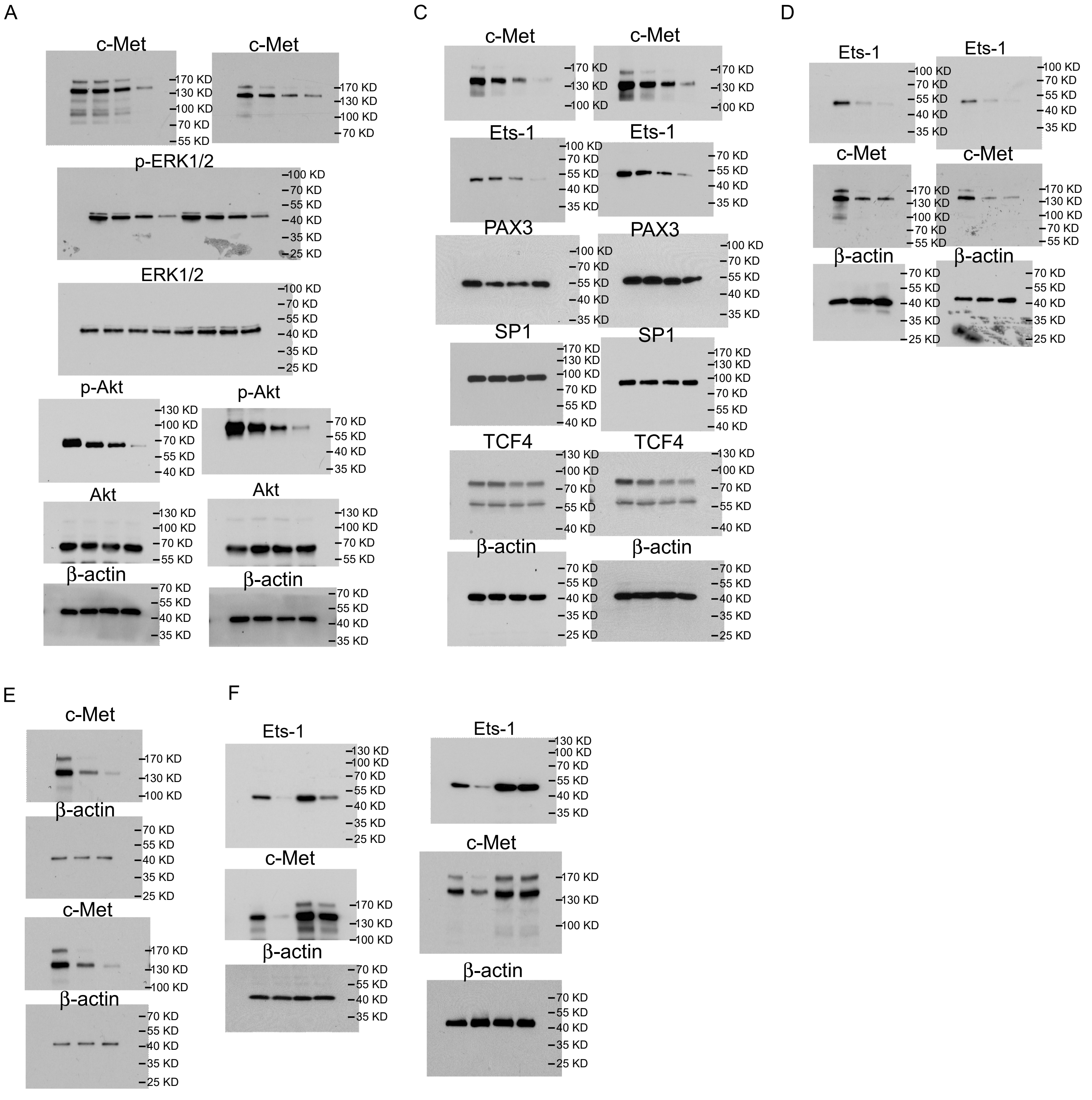

Full gel for Figure 5

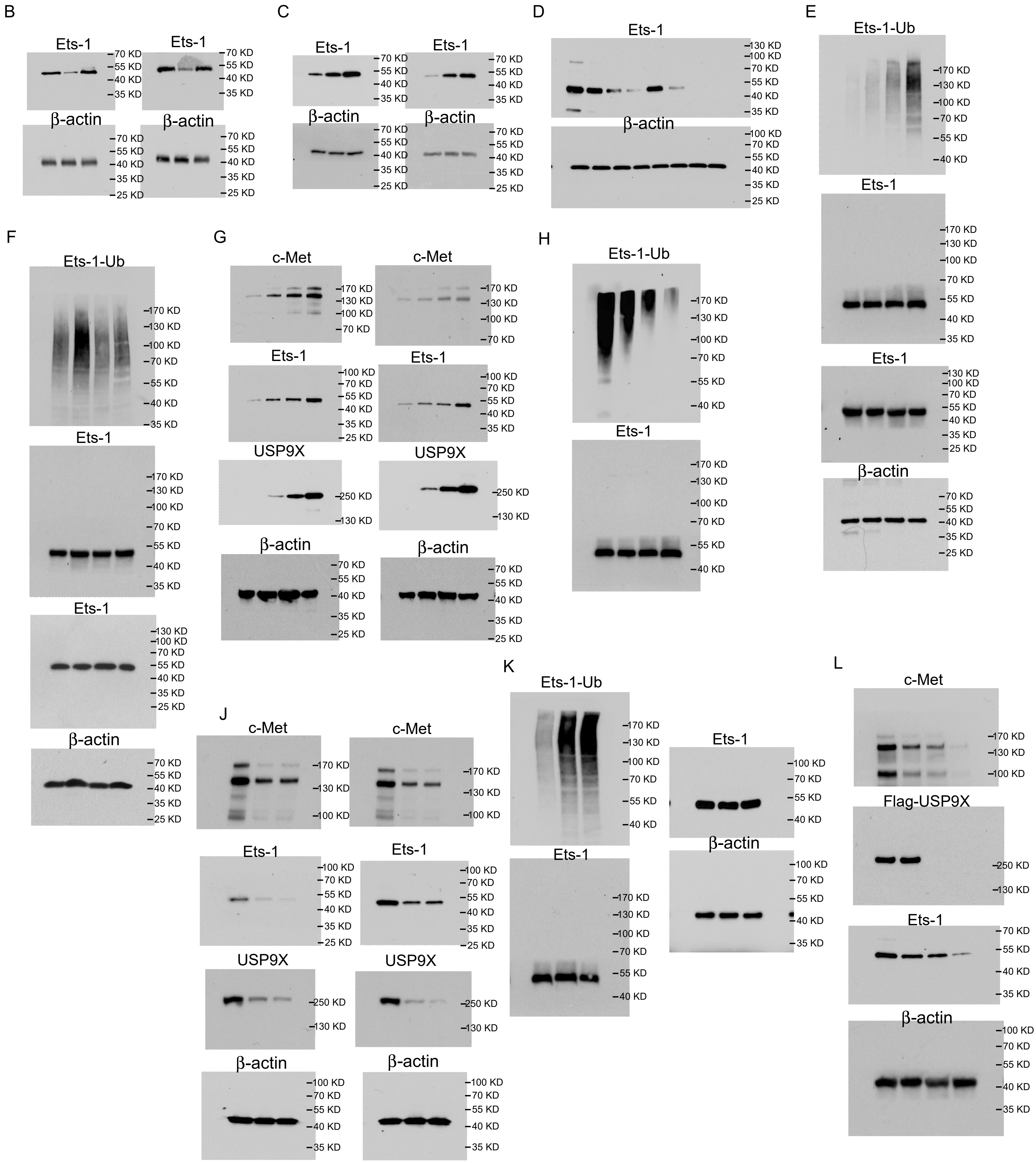

Full gel for Figure 6

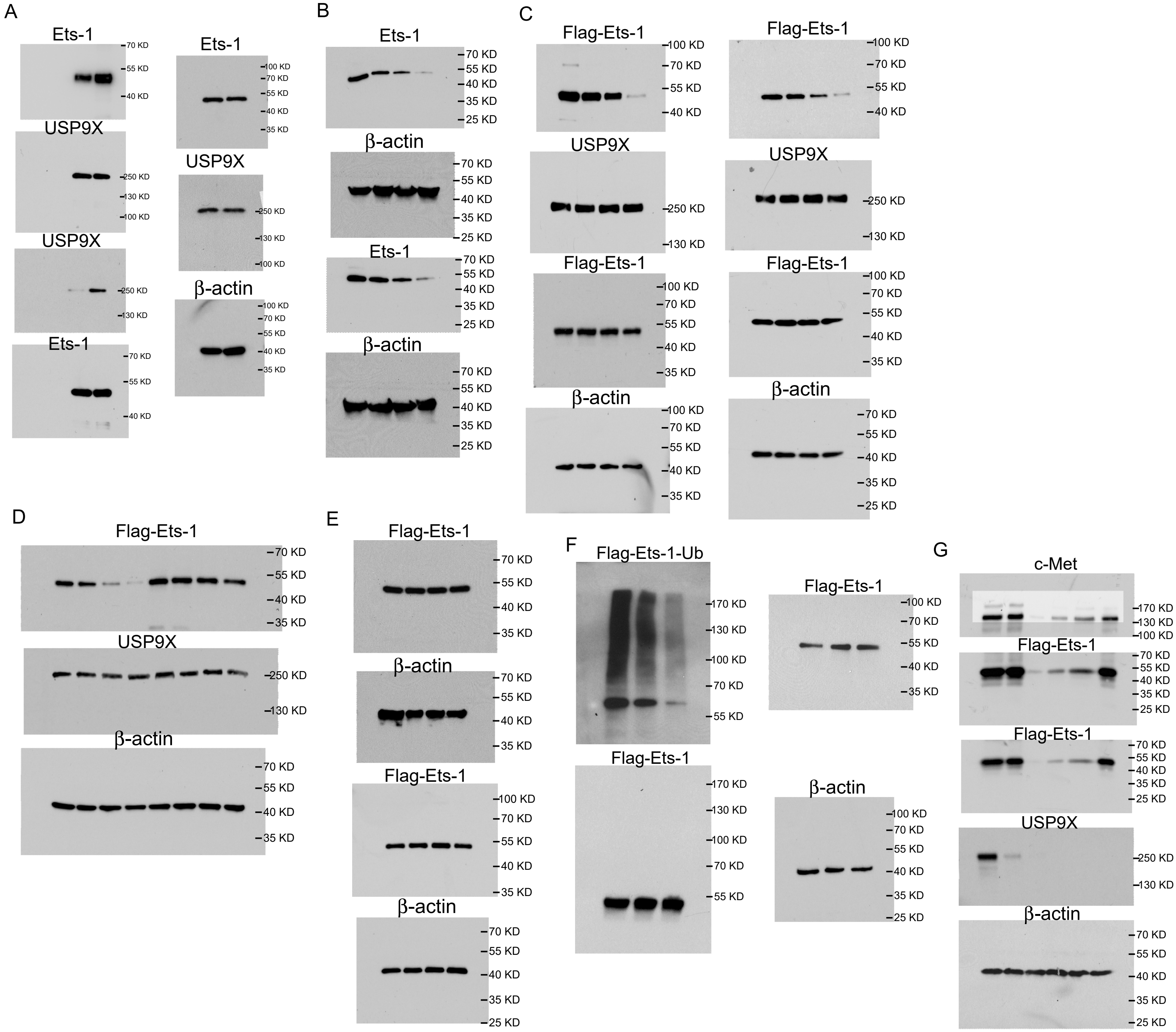

Full gel for Figure 8

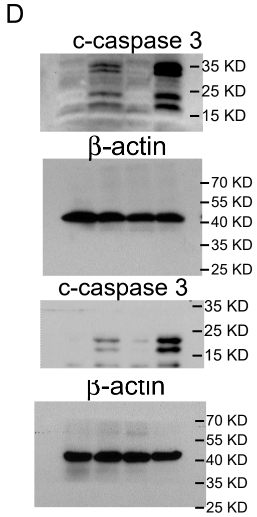

Supplement: Supplementary file 1 — full gel [file 41420_2024_2220_MOESM1_ESM.pdf]
