# Supplementary material for: Xanthohumol overcomes osimertinib resistance via governing ubiquitination-modulated Ets-1 turnover
Source: Cell Death Discov. 2024 Oct 28;10:454. doi: 10.1038/s41420-024-02220-y (PMC11519634; doi:10.1038/s41420-024-02220-y)
Supplement: Supplementary file 2 — Supplementary Figures [file 41420_2024_2220_MOESM2_ESM.docx]

**Supplementary Figure 1**


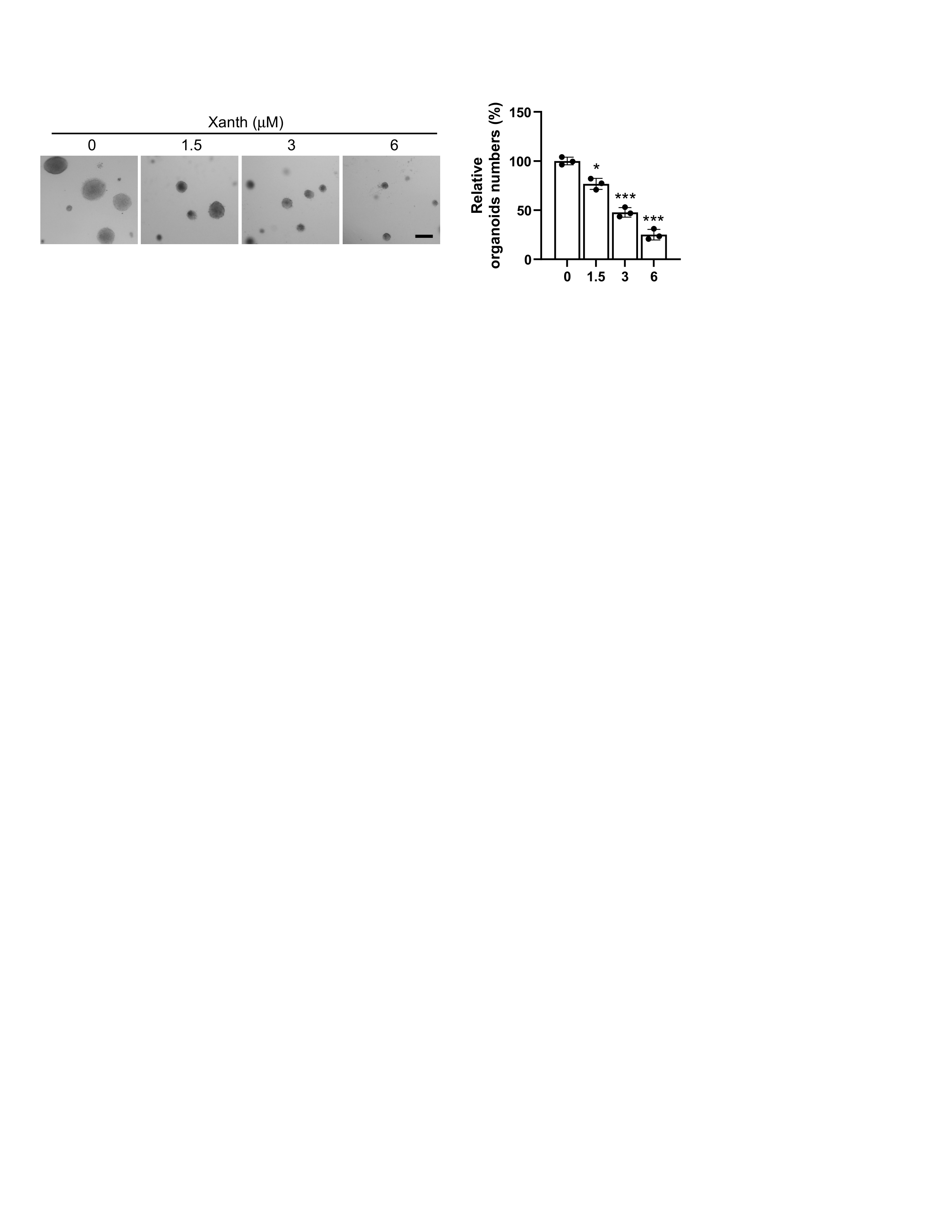


Supplementary Figure 1. The effect of xanthohumol on organoids from primary non-small cell lung cancer patient. Left, representative image; right, qualification. *, p<0.05; ***, p<0.001.

**Supplementary Figure 2**


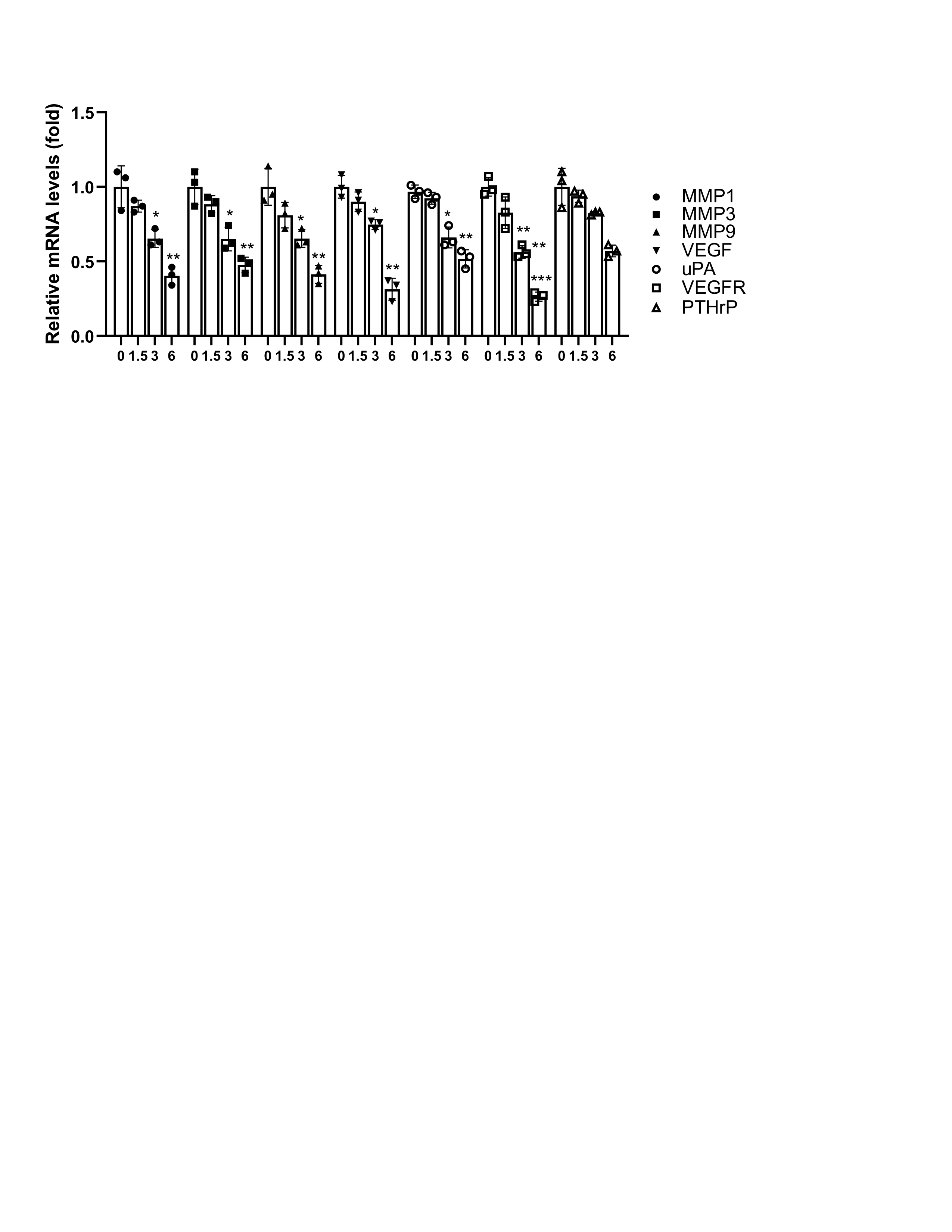


Supplementary Figure 2. qRT-PCR analysis of the mRNA levels of Ets-1 downstream targets, including MMP1, MMP3, MMP9, VEGF, uPA, VEGFR, and PTHrP. *, p<0.05; **, p<0.01; ***, p<0.001.

**Supplementary Figure 3**


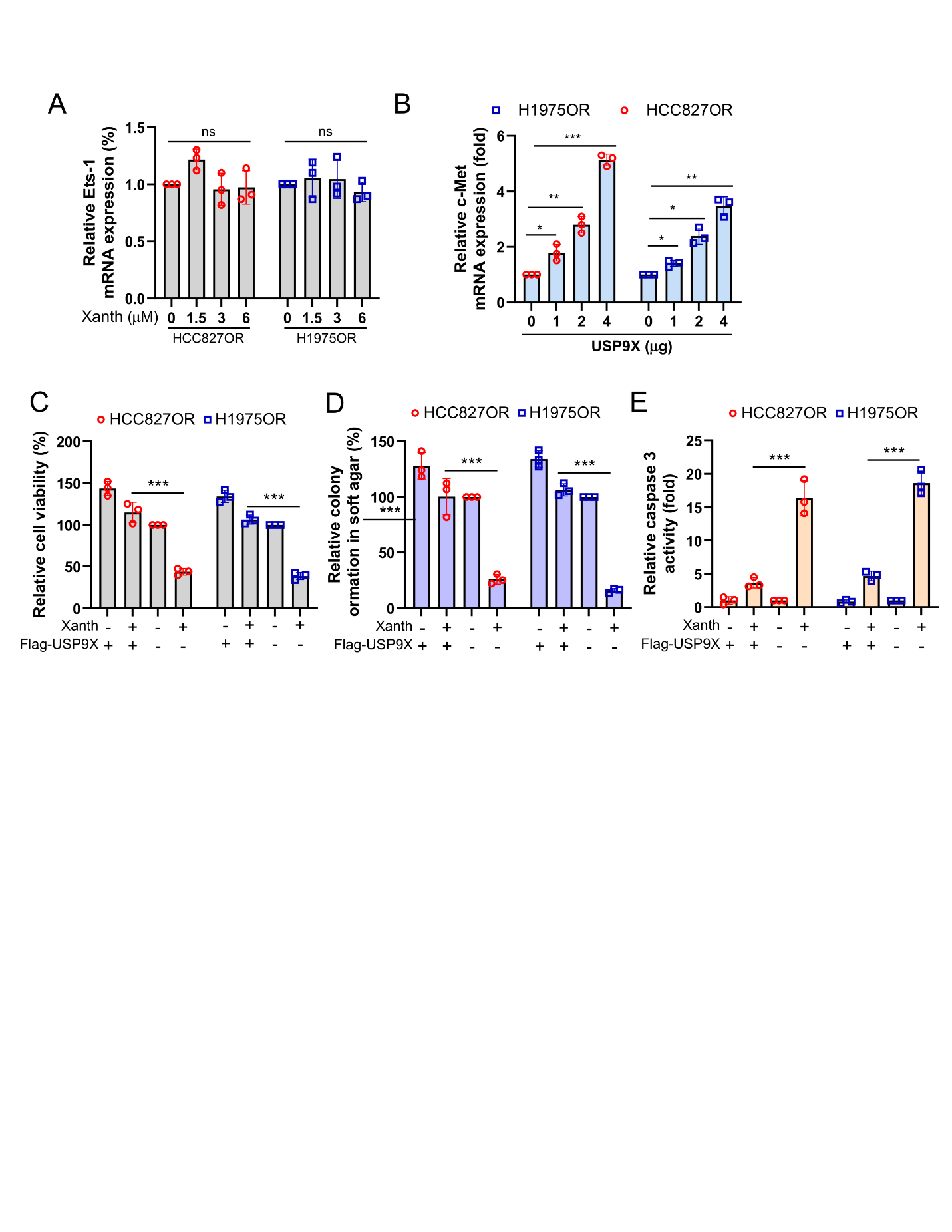


Supplementary Figure 3. A, HCC827OR and H1975OR cells were treated with different concentrations of xanthohumol for 24 h, and RNA was extracted for qPCR to analyze the mRNA level of Ets-1. ns: indicates no statistical significance. B. USP9X plasmids were transfected in HCC827OR and H1975OR cells, and c-Met mRNA expression was detected by qRT-PCR assay. *p<0.05.**p<0.01.***p<0.001. C-E, In HCC827OR cells, xanthohumol treatment, overexpression of USP9X, or co-treatments were given, and cell viability was analyzed by the MTS assay (C); colony forming ability was assayed by soft agar clone formation assay (D); and caspase 3 activity was assayed by caspase 3 activity assay kit (E). ***p<0.001.

**Supplementary Figure 4**


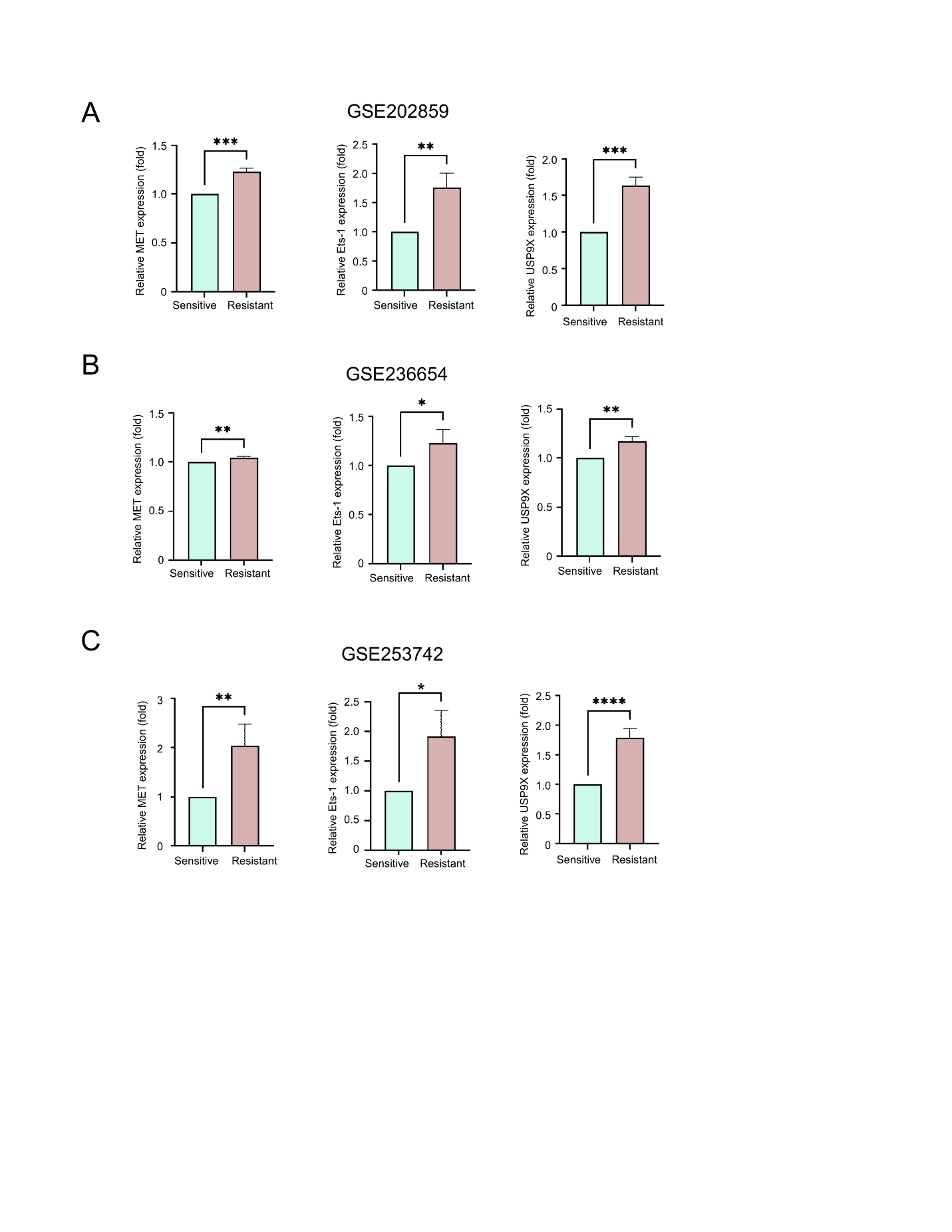


Supplementary Figure 4. A and B, GEO database (GSE202859 (A) and GSE236654 (B)) analysis of the mRNA expression levels of MET, ETS-1, and USP9X in osimertinib-sensitive and -resistant lung cancer cells. C, The expression levels of MET, ETS-1, and USP9X in lung cancer tissues after osimertinib treatment were elevated compared to untreated lung cancer tissues form GEO database (GSE253742). *, p<0.05; **, p<0.01; ***, p<0.001; ****, p<0.0001.

**Supplementary Figure 5**


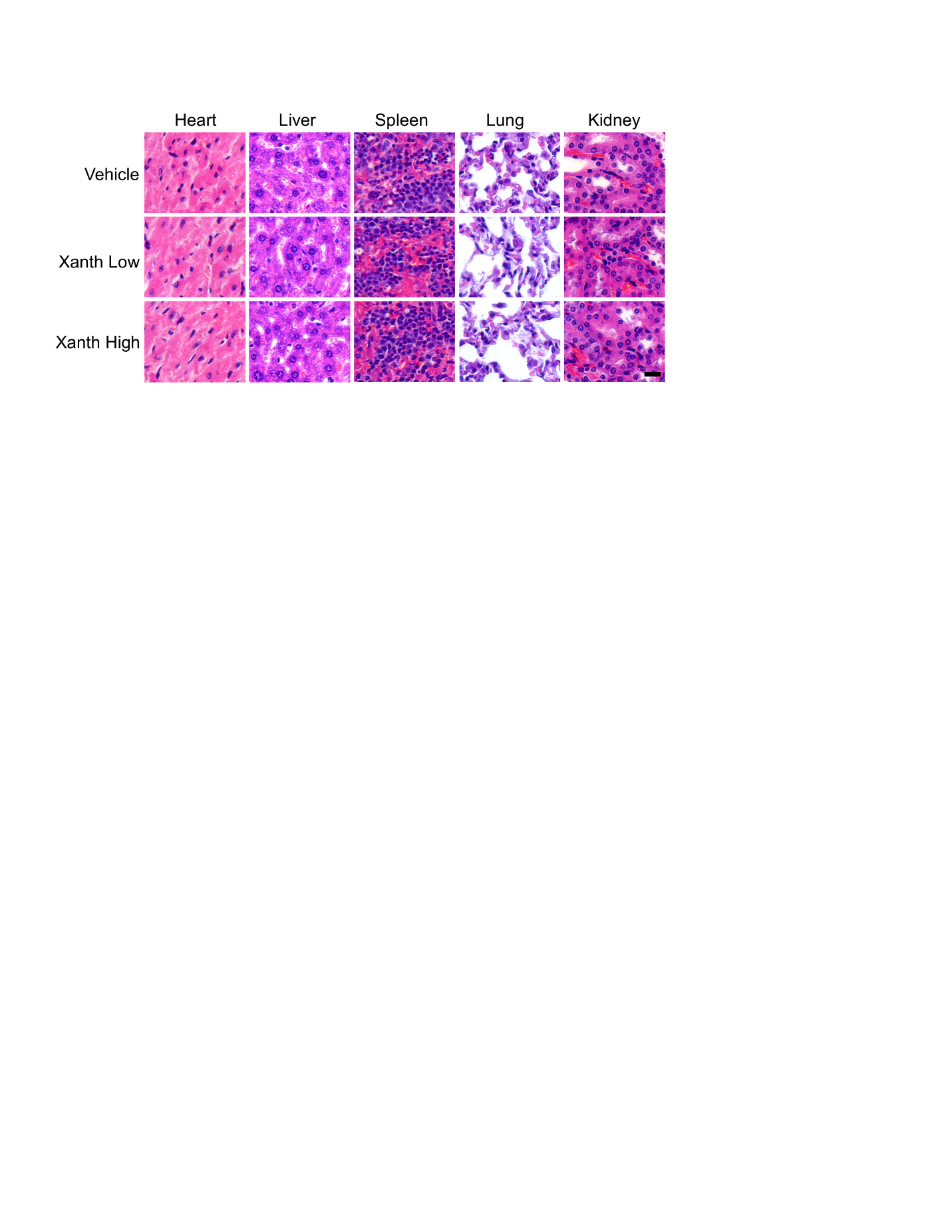


Supplementary Figure 5. The effect of xanthohumol on tumor-bearing mice. HE analyzes the heart, liver, spleen, lung, and kidney of mice treated with different doses of xanthohumol.
